# Impact of sub-setting the data of the main Limousin beef cattle population on the estimates of across-country genetic correlations

**DOI:** 10.1186/s12711-020-00551-9

**Published:** 2020-06-23

**Authors:** Renzo Bonifazi, Jeremie Vandenplas, Jan ten Napel, Kaarina Matilainen, Roel F. Veerkamp, Mario P. L. Calus

**Affiliations:** 1 Animal Breeding and Genomics, Wageningen University & Research, PO Box 338, 6700 AH Wageningen, The Netherlands; 2 Production Systems, Animal Genetics, Natural Resources Institute Finland (Luke), 31600 Jokioinen, Finland

## Abstract

**Background:**

Cattle international genetic evaluations allow the comparison of estimated breeding values (EBV) across different environments, i.e. countries. For international evaluations, across-country genetic correlations (*r*_*g*_) need to be estimated. However, lack of convergence of the estimated parameters and high standard errors of the *r*_*g*_ are often experienced for beef cattle populations due to limited across-country genetic connections. Furthermore, using all available genetic connections to estimate *r*_*g*_ is prohibitive due to computational constraints, thus sub-setting the data is necessary. Our objective was to investigate and compare the impact of strategies of data sub-setting on estimated across-country *r*_*g*_ and their computational requirements.

**Methods:**

Phenotype and pedigree information for age-adjusted weaning weight was available for ten European countries and 3,128,338 Limousin beef cattle males and females. Using a Monte Carlo based expectation–maximization restricted maximum likelihood (MC EM REML) methodology, we estimated across-country *r*_*g*_ by using a multi-trait animal model where countries are modelled as different correlated traits. Values of *r*_*g*_ were estimated using the full data and four different sub-setting strategies that aimed at selecting the most connected herds from the largest population.

**Results:**

Using all available data, direct and maternal *r*_*g*_ (standard errors in parentheses) were on average equal to 0.79 (0.14) and 0.71 (0.19), respectively. Direct-maternal within-country and between-country *r*_*g*_ were on average equal to − 0.12 (0.09) and 0.00 (0.14), respectively. Data sub-setting scenarios gave similar results: on average, estimated *r*_*g*_ were smaller compared to using all data for direct (0.02) and maternal (0.05) genetic effects. The largest differences were obtained for the direct-maternal within-country and between-country *r*_*g*_, which were, on average 0.13 and 0.12 smaller compared to values obtained by using all data. Standard errors always increased when reducing the data, by 0.02 to 0.06, on average. The proposed sub-setting strategies reduced the required computing time up to 22% compared to using all data.

**Conclusions:**

Estimating all 120 across-country *r*_*g*_ that are required for beef cattle international evaluations, using a multi-trait MC EM REML approach, is feasible but involves long computing time. We propose four strategies to reduce computational requirements while keeping a multi-trait estimation approach. In all scenarios with data sub-setting, the estimated *r*_*g*_ were consistently smaller (mainly for direct-maternal *r*_*g*_) and had larger standard errors.

## Background

International genetic evaluations of beef cattle performed by Interbeef allow the comparison of estimated breeding values (EBV) across countries. Current Interbeef evaluations involve up to ten countries, five breeds (Limousin, Charolais, Beef Simmental, Angus, and Hereford), and two trait groups: animal weaning weight (composed of age-adjusted weaning weight) and calving (composed of birth weight and calving ease) (Cromie, personal communication). To estimate international EBV (IEBV), across-country estimated genetic correlations ($r_{g}$) are necessary [1], which in turn require sufficient genetic connections between countries that are usually provided by sires with recorded offspring in more than one country. However, there are two main challenges for the estimation of across-country $r_{g}$ in beef cattle international evaluations: the small number of genetic connections available for the estimation process and the long computing time necessary to obtain them. In beef cattle, although many phenotypes are recorded in both sexes, the number of genetic connections between populations is small due to the limited use of artificial insemination [2]. Such small numbers of genetic connections between populations have been reported since the first Interbeef pilot study [3] and in international evaluations of small dairy breeds [4, 5]. This lack of genetic connections between beef cattle populations makes the estimation of across-country $r_{g}$ more difficult. Furthermore, estimating across-country $r_{g}$ is even more challenging in Interbeef evaluations than in dairy breeds because, in addition to the direct genetic effect, maternal genetic and permanent environment effects are usually included in the model [1, 3].

Estimating across-country $r_{g}$ using all the available data from participating countries would allow the use of all the available genetic connections. However, this has been prohibitive due to computational constraints, and thus, most often, subsets of data are used. To overcome these computational constraints, two main approaches have been used: (1) reduction of the number of populations analysed simultaneously, i.e. country sub-setting, or (2) use of subsets of national submitted data, i.e. within-country data sub-setting [6].

Strategies for country sub-setting reduce the amount of data, but also results in not using all the genetic connections provided by sires with offspring recorded in more than two countries. In turn, not using all the genetic connections may lead to inaccurate estimates of $r_{g}$ and impair the convergence of estimated parameters, resulting in long computing times [6]. Moreover, the resulting across-country $r_{g}$ matrices are very often non-positive definite, as expected for large variance–covariance matrices [7], and require a bending approach, e.g. [8]. The most extreme approach of country sub-setting is the current estimation procedure of Interbeef, which is based on a series of bivariate estimations [9], i.e. by analysing two countries at a time. However, in theory, some of the described shortcomings by Pabiou et al. [9], such as lack of convergence and use of bending, could be overcome by using a multivariate model including all the countries simultaneously.

To date, the application of a within-country data sub-setting approach for a multivariate estimation of $r_{g}$ in Interbeef evaluations has not been fully investigated, mainly because of computational constraints. With such multivariate models and large datasets, traditional restricted maximum likelihood (REML) algorithms are not suitable, which is one of the reasons why Bayesian Gibbs sampling algorithms have been developed and used [10, 11]. Based on García-Cortés et al. [12], Matilainen et al. [13] developed a Monte Carlo based expectation–maximization restricted maximum likelihood (MC EM REML) algorithm that gives the possibility to compute variance components (VC) from a large amount of data using a multi-trait approach, while being more efficient than Gibbs sampling [14].

Thus, our objectives were: (1) to estimate across-country $r_{g}$ for the Limousin Interbeef genetic evaluations by using a multiple trait approach, and (2) to investigate the impact of possible within-country data sub-setting strategies on the estimated $r_{g}$ and associated standard error, and on the required computing time, by taking the low across-country genetic connectedness into account. The within-country data sub-setting strategies aimed at selecting the most connected herds across countries, based on genetic connectedness measures and, for comparison, one strategy used a random selection of herds.

## Methods

### Limousin data and pedigree

Interbeef January 2018 routine evaluation data for age-adjusted weaning weight (AWW) were available for eight Limousin populations, representing ten European countries: Switzerland (CHE), Czech Republic (CZE), Germany (DEU), Denmark, Finland and Sweden (DFS), Spain (ESP), France (FRA), Great Britain (GBR) and Ireland (IRL). The following data edits were applied to the submitted national datasets: (1) animals belonging to contemporary groups (CG) smaller than the defined national minimum size (Table 1), and (2) embryo transfer animals, were removed. The presence of outliers can affect the procedure to estimate variance components both in terms of accuracy of the across-country estimated $r_{g}$ (i.e. standard errors) and of computing time, thus, data that were below or above three phenotypic standard deviations from the phenotypic mean of each population-sex combination were removed. After these edits, individual phenotype records were available for 3,115,598 Limousin males and females, distributed across 19,330 herds.

**Table 1:** Number of age-adjusted weaning weight phenotypes, number of herds, year of birth of recorded animals, and minimum contemporary group size by population

POP^a^	N	%	Herds	YoB^b^	Min CG^c^
CZE	10,500	0.3	121	1991–2017	1
DFS	90,456	2.9	9190	1980–2017	1
ESP	33,152	1.1	188	1989–2011	5
GBR	127,840	4.1	745	1972–2017	5
IRL	20,609	0.7	1304	1975–2017	3
FRA	2,714,368	87.1	6677	1972–2017	2
DEU	88,628	2.8	881	1981–2017	3
CHE	30,045	1.0	224	1993–2017	5
Total	3,115,598	100	19,330	1972–2017	

^a^POP, populations; CZE, Czech Republic; DFS, Denmark, Finland and Sweden; ESP, Spain; GBR, Great Britain; IRL, Ireland; FRA, France; DEU, Germany; CHE, Switzerland

^b^Year of birth

^c^Minimum contemporary group

The numbers of observations available for each population are in Table 1. The FRA population alone represents 87.1% of the observations, followed by the GBR population with 4.1%. DFS and DEU populations represented 2.9 and 2.8% of the observations, respectively. ESP, CHE, IRL and CZE were the smallest populations, each representing 1% or less of the data. Recorded animals were born between 1972 and 2017. FRA and GBR were the only two populations with animals recorded since 1972, whereas submitted records for Spain stopped in 2011 (Table 1). Furthermore, each country adopted different national models for AWW, both in terms of fixed and random effects. National environmental effects for each population are in Additional file 1: Table S1.

Pedigree information for the available data was extracted from the Interbeef international pedigree database using the Interbeef routine workflow, and the following quality controls were performed: all recorded animals without a corresponding record in the pedigree, involved in duplicates and pedigree cycles (i.e. an animal being its own ancestor) were removed. Furthermore, using the RelaX2 software [15], the available pedigree data were pruned to include animals with phenotypes and their ancestors (i.e. using the option “prediction” in RelaX2), without a limit on generation. The final pedigree included 3,431,742 animals, born between 1927 and 2017, and a maximum depth of 19 generations.

### Measure of connectedness

Genetic connections across countries are provided by animals having recorded offspring across two or more populations. First, an analysis was conducted to investigate and quantify the existing common bulls (CB), common dams and common maternal grandsires (CMGS) across-populations. Then, this information was used to compute the following measures of connectedness: coefficients of genetic similarity, coefficient of adjusted number of populations for sires, and the harmonic mean of a sire’s progeny size. Finally, these measures were used to identify the best-connected subsets of data for the estimation of across-country $r_{g}$.

#### Genetic similarity

The concept of genetic similarity between two populations initially proposed by Rekaya et al. [16, 17] has been applied in dairy cattle studies [5, 18] as a measure of connectedness between two countries. We adapted the formula slightly to include sires’ offspring of both sexes to account for the structure of beef cattle data, such that the coefficient of genetic similarity between two populations $a$ and $b$ ($GS_{ab}$) is defined as:

$$GS_{ab} = \frac{{\sum\nolimits_{k = 1}^{2} {\sum\nolimits_{i = 1}^{{CB_{ab} }} {NO_{ik} } } }}{{\sum\nolimits_{k = 1}^{2} {\sum\nolimits_{i = 1}^{{TB_{ab} }} {NO_{ik} } } }},$$

where $CB_{ab}$ is the number of common bulls between populations $a$ and $b$, $TB_{ab}$ is the total number of bulls in populations $a$ and $b$, $NO_{ik}$ is the number of offspring (male and females) of sire $i$ in country $k$ ($k$ = 1, 2).

The coefficient of genetic similarity ranges from 0 to 1 and can be interpreted as the proportion of offspring between two populations that originate from CB. Therefore, the closer the coefficient of genetic similarity between two populations is to 1, the larger is the number of genetic connections between two populations.

#### Balanced offspring distribution (BOD) and adjusted number of populations (AN_POP)

The concept of genetic similarity was extended by Jorjani et al. [6] to take the across-country balanced number of daughters for dairy sires into account by using the coefficient of balanced daughter distribution. We extend this concept to include both male and female offspring, hereafter referred to as the balanced offspring distribution, which is computed for sire $i$ ($BOD_{i}$) as:

$$BOD_{i} = 1 - \frac{{\sum\nolimits_{j = 1}^{{N_{P} }} {\left| {n_{ij} - \bar{n}_{i.} } \right|} }}{{2 \cdot \sum\nolimits_{j = 1}^{{N_{P} }} {n_{ij} } }},$$

where $N_{P}$ is the number of populations in the international genetic evaluation, $n_{ij}$ is the number of offspring of sire $i$ in population $j$, $\bar{n}_{i.}$ is the average number of offspring of sire $i$ across all populations.

Following Jorjani et al. [6], the adjusted number of populations (AN_POP) of sire $i$ was computed for an easier interpretation of the BOD coefficient as:

$$AN\_POP_{i} = N_{P} \cdot BOD_{i} .$$

The BOD coefficient ranges from 0 to 1 and the AN_POP coefficient ranges from 1 to $N_{P}$. For example, a CB with a balanced distribution of recorded offspring across $N$ countries would have an AN_POP coefficient equal to $N$. If the distribution of offspring of CB is not balanced across all $N$ countries, the AN_POP coefficient would be between 1 and $N$.

#### Harmonic mean of a sire’s progeny size

The harmonic mean of a sire’s progeny size across two countries can be used as a measure to identify an unbalanced distribution of offspring. The harmonic mean of the progeny size for sire $i$ ($HM_{i}$) with recorded offspring in two countries can be calculated as:

$$HM_{i} = {2 \mathord{\left/ {\vphantom {2 {\left( {\frac{1}{{N_{1} }} + \frac{1}{{N_{2} }}} \right)}}} \right. \kern-0pt} {\left( {\frac{1}{{N_{1} }} + \frac{1}{{N_{2} }}} \right)}} ,$$

where $N_{1}$ and $N_{2}$ are the progeny sizes of sire $i$ in countries 1 and 2, respectively.

The use of the harmonic mean to measure the unbalanced distribution of offspring can be extended to the herd level as follows:

$$HM_{h} = \mathop \sum \limits_{j = 1}^{{N_{P} }} \mathop \sum \limits_{i = 1}^{{CB_{jh} }} HM_{ijh} ,$$

where $HM_{h }$ is the harmonic mean coefficient for herd $h$, $N_{P}$ is the number of populations in the international genetic evaluation, $CB_{jh}$ is the number of common bulls between population $j$ and herd $h$, $HM_{ijh}$ is the harmonic mean of the $CB_{i}$ progeny size for a common bull $i$ between population $j$ and herd $h$.

### Scenarios

The data sub-setting strategies were focused only on the French Limousin population, since it was the largest national dataset and, therefore, it has a large impact on computing time. Data sub-setting was not applied to the other Limousin populations since it could lead to a relatively large reduction in the number of observations for any of those populations (especially for the smallest ones). In order to minimize variation in data size across different scenarios, and allow a meaningful comparison of computational requirements, the FRA population was reduced in all sub-setting strategies such that the selected amount of data was close to the number of phenotypes for the GBR population, which is the second largest dataset. As a result, the total number of records retained across all populations in any of the data subsets was approximately 0.5 million.

For the subsequent estimation of variance components, we considered different scenarios depending on which FRA records were selected for the analysis, whereas all the records of all other countries were included in all scenarios:

1. Scenario ALL: using the complete dataset, i.e. 3,115,598 AWW records and 3,431,742 animals in the pedigree.
2. Scenario RND: selection of randomly composed groups of FRA herds. FRA herds were randomly divided into 20 subsets. For each subset, the coefficients of genetic similarity with all participating countries were computed. The three subsets with the highest coefficients of genetic similarity were analysed separately. In these subsets of data, 533,816, 521,077 and 556,100 phenotypes were retained, with 706,717, 692,205 and 729,778 animals in the pedigree, respectively.
3. Scenario GSCB: selection of FRA herds based on herd-level coefficients of genetic similarity, defined as the average of the coefficients of genetic similarity computed between herd $h$ and each population $b$ ($GS_{hb}$), with $GS_{hb} = \frac{{\mathop \sum \nolimits_{j} \mathop \sum \nolimits_{i = 1}^{{CB_{hb} }} NO_{ij} }}{{\mathop \sum \nolimits_{j} \mathop \sum \nolimits_{i = 1}^{{TB_{hb} }} NO_{ij} }}$, where $CB_{hb}$ is the number of common bulls between herd $h$ and population $b$, $TB_{hb}$ is the total number of bulls in herd $h$ and population $b$, $NO_{ij}$ is the number of offspring (male and females) of sire $i$ in $j$, with $j = h, b$ (i.e. in herd $h$ or population $b$).
  The final dataset included 506,080 phenotypes and the pruned pedigree included 654,841 animals across all involved countries. The amount of retained data for the FRA population corresponded to 1% of the FRA herds.
4. Scenario GSTOT: selection of FRA herds based on the herd-level coefficient of genetic similarity that includes information from both CB and CMGS (common maternal grand-sires, i.e. maternal grand-sires with grand-offspring in more than one country). Genetic similarity at the herd level was defined as the average of the coefficients of genetic similarity computed between herd $h$ and each population $b$, as $GS_{{TOT_{hb} }} = \left( { GS_{{CB_{hb} }} + GS_{{CMGS_{hb} }} } \right)/2$. $GS_{{CB_{hb} }}$ was the coefficient of genetic similarity computed at the herd level considering CB as defined in Scenario GSCB, and $GS_{{CMGS_{hb} }}$ was the coefficient of genetic similarity computed at the herd level considering CMGS defined as $GS_{{CMGS_{hb} }} = \frac{{\mathop \sum \nolimits_{j} \mathop \sum \nolimits_{i = 1}^{{CMGS_{hb} }} NO_{ij} }}{{\mathop \sum \nolimits_{j} \mathop \sum \nolimits_{i = 1}^{{TMGS_{hb} }} NO_{ij} }}$, where $CMGS_{hb}$ is the number of CMGS between herd $h$ and population $b$, $TMGS_{hb}$ is the number of total maternal grand-sires in herd $h$ and population $b$, $NO_{ij}$ is the number of grand-offspring (male and females) of maternal grand-sire $i$ in $j$, with $j = h, b$ (i.e. in herd $h$ or population $b$).
  The final dataset included 513,969 phenotypes and the pruned pedigree included 663,127 animals across all involved countries. The amount of retained data for the FRA population corresponded to the top 1% of the FRA herds ranked on their $GS_{TOT}$ coefficient.
5. Scenario HM: selection of FRA herds based on the harmonic mean of sires’ progeny size. Based on the harmonic means computed at the herd level, FRA herds were selected until the FRA population was reduced to about 0.5 million records. The final dataset included 502,716 phenotypes, with a pruned pedigree of 649,081 animals across all involved countries.

When data reduction was applied to the FRA population (i.e. all scenarios except Scenario ALL), the RelaX2 software was used for pedigree pruning with the following options: “prediction” pruning method and no generation limit.

### Model and software

In all scenarios, variance component estimation (VCE) was performed using an animal model accounting for across-country interaction (AMACI) [1]. The AMACI model accounts for country-specific fixed and random effects by fitting for each country their national model. The AMACI model, currently used for Interbeef routine evaluations, is equivalent to a multi-trait animal model with maternal effects, where each population is modelled as a different trait:

$$\begin{aligned} & \left[ {\begin{array}{*{20}c} {{\mathbf{y}}_{1} } \\ {{\mathbf{y}}_{2} } \\ {\begin{array}{*{20}c} \vdots \\ {{\mathbf{y}}_{8} } \\ \end{array} } \\ \end{array} } \right] = \left[ {\begin{array}{*{20}c} {{\mathbf{X}}_{1} } & \cdots & 0 \\ \vdots & \ddots & \vdots \\ 0 & \cdots & {{\mathbf{X}}_{8} } \\ \end{array} } \right] \left[ {\begin{array}{*{20}c} {\begin{array}{*{20}c} {{\mathbf{b}}_{1} } \\ \vdots \\ \end{array} } \\ {{\mathbf{b}}_{8} } \\ \end{array} } \right] \\ & \quad + \left[ {\begin{array}{*{20}c} {\begin{array}{*{20}c} {{\mathbf{C}}_{1} } \\ \end{array} } & 0 & 0 & 0 \\ 0 & {{\mathbf{C}}_{2} } & 0 & 0 \\ 0 & 0 & {{\mathbf{C}}_{3} } & 0 \\ 0 & 0 & 0 & {{\mathbf{C}}_{4} } \\ \end{array} } \right] \left[ {\begin{array}{*{20}c} {\begin{array}{*{20}c} {{\mathbf{r}}_{1} } \\ {{\mathbf{r}}_{2} } \\ \end{array} } \\ {{\mathbf{r}}_{3} } \\ {{\mathbf{r}}_{4} } \\ \end{array} } \right] + \left[ {\begin{array}{*{20}c} {{\mathbf{Z}}_{1} } & \cdots & 0 \\ \vdots & \ddots & \vdots \\ 0 & \cdots & {{\mathbf{Z}}_{8} } \\ \end{array} } \right] \left[ {\begin{array}{*{20}c} {\begin{array}{*{20}c} {{\mathbf{u}}_{1} } \\ \vdots \\ \end{array} } \\ {{\mathbf{u}}_{8} } \\ \end{array} } \right] \\ & \quad + \left[ {\begin{array}{*{20}c} {{\mathbf{W}}_{1} } & \cdots & 0 \\ \vdots & \ddots & \vdots \\ 0 & \cdots & {{\mathbf{W}}_{8} } \\ \end{array} } \right] \left[ {\begin{array}{*{20}c} {\begin{array}{*{20}c} {{\mathbf{m}}_{1} } \\ \vdots \\ \end{array} } \\ {{\mathbf{m}}_{8} } \\ \end{array} } \right] + \left[ {\begin{array}{*{20}c} {{\mathbf{P}}_{1} } & \cdots & 0 \\ \vdots & \ddots & \vdots \\ 0 & \cdots & {{\mathbf{P}}_{7} } \\ \end{array} } \right] \left[ {\begin{array}{*{20}c} {\begin{array}{*{20}c} {{\mathbf{pe}}_{1} } \\ \vdots \\ \end{array} } \\ {{\mathbf{pe}}_{7} } \\ \end{array} } \right] + \left[ {\begin{array}{*{20}c} {\begin{array}{*{20}c} {{\mathbf{e}}_{1} } \\ \vdots \\ \end{array} } \\ {{\mathbf{e}}_{8} } \\ \end{array} } \right], \\ \end{aligned}$$

where ${\mathbf{y}}_{i}$ is the vector of observations for population $i$; ${\mathbf{b}}_{i}$ is the vector of fixed effects for population $i$; ${\mathbf{r}}_{i}$ is the vector of random environmental effects for population $i$; ${\mathbf{u}}_{i}$ is the vector of random additive genetic (direct) effects; ${\mathbf{m}}_{i}$ is the vector of random maternal (indirect) additive genetic effects; ${\mathbf{pe}}_{i}$ is the vector of random maternal permanent environmental effects (provided by the dam); ${\mathbf{e}}_{i}$ is the vector of random residual effects. ${\mathbf{X}}$ and ${\mathbf{C}}$ are incidence matrices linking records to fixed and random environmental effects, respectively. ${\mathbf{Z}}$, ${\mathbf{W}}$, and ${\mathbf{P}}$ are incidence matrices linking records to the animal, maternal genetic and maternal permanent environmental effects, respectively.

The four random environmental effects refer to the national effects of herd-year-season in CZE, herd-year in DEU and CHE, and sire-herd in CHE (see Additional file 1: Table S1). Furthermore, a maternal permanent environmental effect was not fit for the DEU population in the national evaluation (Ruten, personal communication). Because the international model follows the national evaluation models, DEU was the only population without a maternal permanent environmental effect in the AMACI model (see Additional file 1: Table S1).

It is assumed that:

$$var\left[ {\begin{array}{*{20}c} {{\mathbf{u}}_{1} } \\ {{\mathbf{u}}_{2} } \\ \vdots \\ {{\mathbf{u}}_{8} } \\ {{\mathbf{m}}_{1} } \\ {{\mathbf{m}}_{2} } \\ \vdots \\ {{\mathbf{m}}_{8} } \\ \end{array} } \right] = \left[ {\begin{array}{*{20}c} {\sigma^{2}_{u1} } & {} & {} & {} & {} & {} & {} & {} \\ {\sigma_{u1,u2} } & {\sigma^{2}_{u2} } & \ldots & {} & {} & {Sym} & {} & {} \\ \vdots & \vdots & \ddots & {} & {} & {} & {} & {} \\ {\sigma_{u1,u8} } & {\sigma_{u1,u8} } & \ldots & {\sigma^{2}_{u8} } & {} & {} & {} & {} \\ {\sigma_{u1,m1} } & {\sigma_{u2,m1} } & \ldots & {\sigma^{2}_{u8,m1} } & {\sigma^{2}_{m1} } & {} & {} & {} \\ {\sigma_{u1,m2} } & {\sigma_{u2,m2} } & \ldots & {\sigma^{2}_{u8,m2} } & {\sigma^{2}_{m1,m2} } & {\sigma^{2}_{m2} } & {} & {} \\ \vdots & \vdots & \ldots & \vdots & \vdots & \vdots & \ddots & {} \\ {\sigma_{u1,m8} } & {\sigma_{u2,m8} } & \ldots & {\sigma^{2}_{u8,m8} } & {\sigma^{2}_{m1,m8} } & {\sigma^{2}_{m2,m8} } & \ldots & {\sigma^{2}_{m8} } \\ \end{array} } \right] \otimes {\mathbf{A,}}$$

$$var\left( {\mathbf{r}} \right) = \begin{array}{*{20}c} 4 \\ \oplus \\ {i = 1} \\ \end{array} {\mathbf{I}}_{ni} \cdot \sigma_{ri}^{2} ,$$

$$var\left( {{\mathbf{pe}}} \right) = \begin{array}{*{20}c} 7 \\ \oplus \\ {i = 1} \\ \end{array} {\mathbf{I}}_{ni} \cdot \sigma_{pei}^{2} ,$$

$$var\left( {\mathbf{e}} \right) = \begin{array}{*{20}c} 8 \\ \oplus \\ {i = 1} \\ \end{array} {\mathbf{I}}_{ni} \cdot \sigma_{ei}^{2} ,$$

where, $\sigma^{2}_{{u_{i} }}$ is the direct additive genetic variance for population $i$, $\sigma^{2}_{{m_{i} }}$ is the maternal additive genetic variance for population $i$, $\sigma_{{u_{i} , m_{i} }}$ is the additive genetic covariance between direct and maternal effect for population $i$, $\sigma_{{u_{i} , u_{j} }}$ is the additive genetic covariance between populations $i$ and $j$, $\sigma_{{u_{i} , m_{j} }}$ is the additive genetic covariance between the direct effect for population $i$ and the maternal effect for population $j$, $\sigma_{{r_{i} }}^{2}$ is the random environmental variance for population $i$, $\sigma_{{pe_{i} }}^{2}$ is the permanent environmental variance for population $i$, $\sigma_{{e_{i} }}^{2}$ is the residual error variance for population $i$, ${\mathbf{A}}$ is the numerator relationship matrix, ${\mathbf{I}}$ is the identity matrix, $\oplus$ indicates the matrix direct sum of $i$ diagonal matrices, and $\otimes$ indicates the Kronecker product. Permanent environmental covariances between countries were fixed to 0 since only 697 dams had offspring in more than one country.

All analyses for VCE were conducted using the MC EM REML algorithm as implemented in the MiX99 software [19]. The MC EM REML algorithm performs two main steps within each REML round. In the first step, best linear unbiased prediction (BLUP) estimates of random effects are obtained from the real data. In the second step, BLUP estimates of random effects are obtained from repeatedly simulated data based on the model and a given set of VC. Successively, via MC EM REML, VC are estimated using the sum of the squares of estimated random effects obtained from the real data, and the prediction error variances obtained from the simulated data. For both the real and simulated data, the preconditioned conjugate gradient (PCG) algorithm is used to obtain solutions of the mixed model equations. The PCG convergence criterion is defined as the square root of the relative difference between solutions of consecutive PCG iteration rounds. In the REML round step, the convergence criterion for VCE is defined by using a linear regression of the estimated VC over the last REML rounds. When the linear regression slope is smaller than a given value, convergence is reached (for more details see Matilainen et al. [13]).

The VCE estimated via MC EM REML was standardized for all scenarios using: (1) a maximum of 1000 PCG iterations, (2) a convergence criterion for PCG iterations equal to 10^−5^, (3) one simulated dataset for each REML round, and (4) a convergence criterion of 10^−9^ for the VCE. In all scenarios, the provided set of starting values were the most recent estimates available and currently in use for the 2018 Limousin Interbeef routine evaluations.

Approximated standard errors (SE) of the estimated VC can be obtained in an additional MC EM REML round [20]. This additional MC EM REML round was performed using the same setting as described above for VCE, but with 500 simulated datasets and no limit for the maximum number of PCG iterations. The same settings were used in all scenarios. Approximated standard errors of across-country $r_{g}$ were calculated from the obtained information matrix as described by Klei and Tsuruta [21].

## Results

First, we present the results for the assessment of the available genetic connections in the whole dataset, followed by the estimated $r_{g}$ in each scenario and their computational requirements.

### Measures of connectedness

#### Common bulls and common maternal grand sires

We assessed the available genetic connections between countries by quantifying the number of recorded offspring of CB. The number of CB varied with the country combination, with a minimum of 44 CB between ESP-CZE and a maximum of 396 for FRA-GBR (Table 2). The average number of CB between two populations was 143.6. The number of sires used within a country varied considerably, with a minimum of 554 bulls for CZE, and a maximum of 57,784 bulls for FRA, which reflects the differences in the population sizes of participating countries.

**Table 2:** Total number of bulls used within population (diagonal), number of common bulls (above diagonal) and genetic similarity coefficients (below diagonal) between populations

POP^a^	CZE	DFS	ESP	GBR	IRL	FRA	DEU	CHE
CZE	554	65	44	67	64	157	101	63
DFS	0.06	4375	76	109	94	171	143	73
ESP	0.07	0.06	1188	97	78	358	105	71
GBR	0.04	0.05	0.04	5486	239	396	125	72
IRL	0.14	0.07	0.12	0.15	2073	200	120	65
FRA	0.11	0.13	0.13	0.13	0.12	57,784	339	342
DEU	0.06	0.06	0.06	0.04	0.07	0.15	4366	188
CHE	0.12	0.06	0.08	0.04	0.10	0.13	0.11	1699

^a^POP, population; CZE, Czech Republic; DFS, Denmark, Finland and Sweden; ESP, Spain; GBR, Great Britain; IRL, Ireland; FRA, France; DEU, Germany; CHE, Switzerland

Since CB can have recorded offspring in more than one bivariate country-combination, hereafter we will use the term “unique CB” to indicate an individual CB. The total number of unique CB in the available dataset was equal to 1436. Of these unique CB, 1053 (73.4%) had offspring in two populations, while 12.4, 5.2, 4.4, 2.2, 1.7 and 1.2% had offspring in three up to all eight populations, respectively. Moreover, the distribution of CB by number of connecting populations and by country of origin (Table 3) showed that the majority of CB were from France (82.5%), followed by Germany (6.3%), Great Britain (5.7%), Denmark (2.0%) and Ireland (1.81%). Finally, sires that connected four or more populations were mostly French bulls (Table 3).

**Table 3:** Distribution of common bulls per country of first registration and across different numbers of connected populations

COU^a^	Number of connected populations							
	2	3	4	5	6	7	8	Sum
CAN	5	1	1					7
CHE	4							4
CZE	3							3
DEU	71	11	4	3	2			91
DNK	25	2	1	1				29
ESP	1	1						2
FRA	861	139	61	58	29	20	17	1185
GBR	57	19	5	1				82
IRL	21	4	1					26
LUX	3							3
NOR	1							1
SWE		1						1
USA	1		1					2
Sum	1053	178	74	63	31	20	17	1436

^a^COU, country of first registration; CAN, Canada; CHE, Switzerland; CZE, Czech Republic; DEU, Germany; DNK, Denmark; ESP, Spain; FRA, France; GBR, Great Britain; IRL, Ireland; LUX, Luxemburg; NOR, Norway; SWE, Sweden; USA, United States of America

**Fig. 1:**
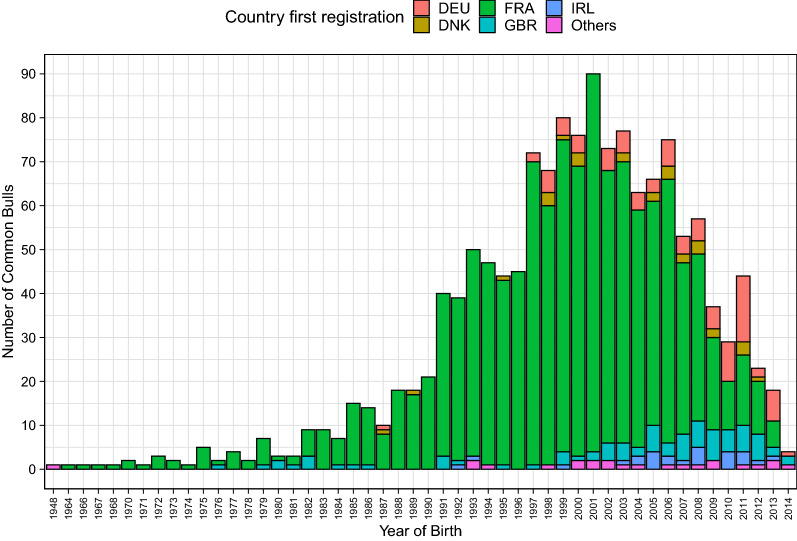
Distribution of year of birth of common bulls and their country of first registration. DEU, Germany; DNK, Denmark; FRA, France; GBR, Great Britain; IRL, Ireland

The distribution of the number of CB per year of birth and country of first registration reflects the exchange of genetic material across the analysed populations (Fig. 1). Figure 1 indicates that consistent genetic connections between countries started with the use of CB born during the 1970s and 1980s. In addition, the use of sires born in more recent years reflects the use of more recent genetic material, with the majority of the CB born after the 1990s, and with the highest frequency of year of birth of CB being in 2001 (Fig. 1). Furthermore, French sires represented a large proportion of CB in each year, with sires from Germany, Ireland and Great Britain becoming more frequently used at the international level during the last decade.

Common maternal grand-sires can also provide valuable genetic connections [1, 3], particularly for the estimation of the maternal and direct-maternal across-country $r_{g}$. Table 4 shows the distribution of CMGS per number of connected populations. Of the total 3828 unique CMGS, 79.4% had recorded grand-offspring in only two populations, whereas there is an inversely proportional relationship between number of CMGS and number of connected populations with 13.2, 3.1, 1.9, 1.2, 0.7 and 0.5% of CMGS connecting three up to all eight populations, respectively. Furthermore, 25.5% of the CMGS are also CB (Table 4).

**Table 4:** Distribution of common maternal grand-sires (CMGS) with grand-offspring in two or more populations and number of CMGS that are also common bulls (CB)

Connected POP	CMGS	CMGS also CB	
		Yes	No
2	3040	552	2488
3	507	204	303
4	119	76	43
5	72	57	15
6	46	42	4
7	25	24	1
8	19	19	0
Sum	3828	974	2854

#### Genetic similarity

Coefficients of genetic similarity between populations are in Table 2. Jorjani [18] used a coefficient of genetic similarity of 0.06 as a threshold to divide Ayrshire bull populations into two country groups. However, in the literature, we found no other clear thresholds for the coefficient of genetic similarity to define the level of connectedness between two populations. Therefore, we defined three arbitrary thresholds for the coefficient of genetic similarity: a low, medium and high level of connectedness for coefficients of genetic similarity lower than 0.05, between 0.05 and 0.10, and higher than 0.10, respectively.

Overall, we observed a medium level of across-country connections when all the data were considered, with an average coefficient of genetic similarity of 0.09 across populations. All the populations showed a high level of connectedness with FRA (> 0.10), which reflects the high proportion of French CB. Moreover, the IRL population had a high level of connections with all countries except with DFS and DEU. As a result, IRL and FRA were the two populations with the highest average coefficient of genetic similarity with other countries (0.11 and 0.13, respectively). GBR showed the lowest bivariate connections (GS < 0.05) with CZE, ESP, DEU, and CHE.

#### Balanced offspring distribution (BOD) and adjusted number of populations (AN_POP)

The balanced offspring distribution and the adjusted number of population coefficients can be considered as two quantitative measures of the same quantity, i.e. balance in sires’ offspring records across country. Table 5 reports the AN_POP and BOD distribution for CB. Among all CB, none had a balanced distribution across all eight populations, resulting in all sires having an AN_POP smaller than 8 and a maximum AN_POP of 5 for a single CB. The majority of the CB (> 65%) had an AN_POP smaller than 2. In addition, 23.1% of the CB had an AN_POP of 2 and only 11.9% of the total CB had AN_POP larger than 2. Since the AN_POP coefficients are computed as a function of the BOD coefficients, their distributions are similar.

**Table 5:** Average number of populations (AN_POP) and balanced offspring distribution (BOD) coefficients for common bulls (CB)

AN_POP	BOD	Number of CB
Balanced		
= 2	= 0.25	332
= 3	= 0.375	18
= 4	= 0.5	0
= 5	= 0.625	1
Unbalanced		
> 1–1.999	> 0.125–0.25	933
> 2–2.999	> 0.25–0.375	104
> 3–3.999	> 0.375–0.5	43
> 4–4.999	> 0.5–0.625	5
Sum (all CB)		
> 1	> 0.125	1436

### Estimated genetic correlations in different scenarios

Modelling the countries as different traits in international evaluations, as in the AMACI model, allows the genetic correlations between countries to be lower than 1, which accounts for genotype-by-environment interactions and possible differences in phenotypic distribution, trait and national model definition of the AWW. Descriptive statistics for each population-sex combination highlight differences in phenotypic mean for AWW across populations (see Additional file 1: Table S2). These differences may be associated with a variation in trait definition across countries and, in particular, with the adjustment criteria applied (see Additional file 1: Table S3). Although an improved harmonization of traits across countries is desirable, it does not remove the need to model each country as a separate trait.

Using different approaches to select the data leads to different subsets of data for the FRA population. First, we present the estimated $r_{g}$ for Scenario ALL, and second, we provide a description of the data selected and the result yielded in each sub-setting scenario.

Results of the across-country estimated $r_{g}$ and approximated standard errors (SE) when using all the data (Scenario ALL) are in Table 6. The average across-country $r_{g}$ for the direct genetic effect was equal to 0.79 and ranged from 0.62 (DEU-IRL) to 0.94 (DEU-DFS). The average across-country $r_{g}$ for the maternal effect was equal to 0.71 and ranged from 0.65 (CHE-IRL) to 0.87 (FRA-GBR). The average estimated direct-maternal within-country $r_{g}$ was equal to − 0.12 and ranged from − 0.33 for FRA to 0.40 for CHE. Direct-maternal between-country $r_{g}$ were on average equal to 0 and ranged from − 0.14 (GBR-FRA) to 0.14 (GBR-CZE). For the CHE population, most of the direct-maternal between-country $r_{g}$ were positive (average of 0.06), whereas for the FRA population most of the direct-maternal $r_{g}$ were negative (average of − 0.06). SE of the estimated $r_{g}$ in Scenario ALL were on average equal to 0.14 for the direct $r_{g}$ (ranging from 0.06 to 0.22), and 0.19 for the maternal $r_{g}$ (ranging from 0.07 to 0.33). SE of direct-maternal within-country and between-country $r_{g}$ were on average equal to 0.09 (ranging from 0.02 to 0.16), and 0.14 (ranging from 0.06 to 0.23), respectively. The DEU-FRA combination always had the largest SE of estimated $r_{g}$ and the ESP-CHE combination had the smallest.

**Table 6:** Scenario ALL—heritabilities (italic characters on the diagonal), estimated genetic correlations (below diagonal) and standard errors of estimated correlations (above diagonal), for direct and maternal genetic effects

	Direct								Maternal							
	CZE	DFS	ESP	GBR	IRL	FRA	DEU	CHE	CZE	DFS	ESP	GBR	IRL	FRA	DEU	CHE
Direct																
CZE	*0.24*	0.16	0.21	0.15	0.19	0.12	0.14	0.22	0.10	0.15	0.20	0.16	0.16	0.10	0.12	0.19
DFS	0.87	*0.30*	0.16	0.10	0.13	0.07	0.10	0.15	0.13	0.06	0.14	0.11	0.12	0.06	0.09	0.16
ESP	0.74	0.77	*0.13*	0.17	0.20	0.14	0.17	0.22	0.20	0.18	0.16	0.16	0.16	0.10	0.15	0.22
GBR	0.71	0.82	0.94	*0.29*	0.14	0.06	0.10	0.18	0.15	0.12	0.15	0.06	0.11	0.06	0.11	0.18
IRL	0.83	0.76	0.87	0.91	*0.35*	0.11	0.13	0.21	0.16	0.15	0.18	0.11	0.14	0.09	0.12	0.20
FRA	0.76	0.89	0.77	0.82	0.76	*0.29*	0.06	0.13	0.09	0.08	0.10	0.08	0.10	0.02	0.06	0.12
DEU	0.76	0.94	0.76	0.77	0.62	0.81	*0.24*	0.14	0.13	0.11	0.16	0.13	0.13	0.06	0.05	0.15
CHE	0.85	0.81	0.76	0.71	0.70	0.70	0.70	*0.12*	0.19	0.18	0.23	0.20	0.19	0.11	0.14	0.10
Maternal																
CZE	− 0.12	0.04	0.07	0.12	− 0.01	− 0.10	0.08	0.01	*0.18*	0.20	0.26	0.21	0.22	0.14	0.16	0.27
DFS	− 0.05	− 0.14	0.02	− 0.01	− 0.02	− 0.11	− 0.07	− 0.01	0.68	*0.14*	0.23	0.18	0.19	0.11	0.14	0.24
ESP	0.03	0.09	− 0.22	− 0.08	− 0.09	− 0.05	0.05	0.02	0.67	0.68	*0.07*	0.24	0.25	0.15	0.21	0.33
GBR	0.14	0.06	− 0.03	− 0.10	− 0.03	− 0.14	0.07	0.08	0.79	0.69	0.70	*0.07*	0.18	0.12	0.16	0.26
IRL	− 0.03	0.07	− 0.06	− 0.05	− 0.19	− 0.12	0.12	0.11	0.69	0.68	0.81	0.72	*0.17*	0.16	0.16	0.24
FRA	− 0.02	− 0.05	− 0.03	− 0.06	− 0.09	− 0.33	− 0.01	0.08	0.85	0.69	0.71	0.87	0.82	*0.09*	0.07	0.17
DEU	− 0.02	− 0.09	− 0.03	− 0.01	0.06	− 0.10	− 0.24	0.09	0.68	0.68	0.67	0.69	0.68	0.69	*0.20*	0.20
CHE	0.12	0.11	0.07	0.08	0.03	− 0.05	0.06	0.40	0.73	0.68	0.67	0.66	0.65	0.77	0.66	*0.05*

Population: CZE, Czech Republic;DFS, Denmark, Finland and Sweden; ESP, Spain; GBR, Great Britain; IRL, Ireland; FRA, France; DEU, Germany; CHE, Switzerland

Table 7 provides a summary of the comparison between each sub-setting scenario (RND, GSCB, GSTOT and HM) and Scenario ALL, for the across-country estimated $r_{g}$ and SE. Complete comparisons are in Additional file 1: Tables S4, S5, S6, and S7.

**Table 7:** Summary statistics for estimated across-country genetic correlations (*r*_*g*_) and their standard errors (SE), for the direct, maternal and direct-maternal effect (within and between-country) in ALL versus each sub-setting scenario (RND, GSCB, GSTOT, HM)

Scenario^a^	Direct			Maternal			Direct-maternal					
							Within-country			Between-country		
	Average	Min	Max	Average	Min	Max	Average	Min	Max	Average	Min	Max
Genetic correlations												
ALL	0.79	0.62	0.94	0.71	0.65	0.87	− 0.12	− 0.33	0.40	0.00	− 0.14	0.14
Difference^b^ in *r*_*g*_												
RND	− 0.02	− 0.04	− 0.01	− 0.04	− 0.07	− 0.02	− 0.12	− 0.17	− 0.04	− 0.11	− 0.16	− 0.06
GSCB	− 0.02	− 0.03	0.00	− 0.05	− 0.08	− 0.03	− 0.13	− 0.17	− 0.04	− 0.11	− 0.17	− 0.03
GSTOT	− 0.02	− 0.03	0.00	− 0.05	− 0.08	− 0.03	− 0.13	− 0.18	− 0.05	− 0.12	− 0.17	− 0.04
HM	− 0.02	− 0.04	0.00	− 0.06	− 0.09	− 0.03	− 0.11	− 0.17	− 0.03	− 0.10	− 0.17	− 0.02
Standard errors												
ALL	0.14	0.06	0.22	0.19	0.07	0.33	0.09	0.02	0.16	0.14	0.06	0.23
Difference^b^ in SE												
RND	0.03	0.01	0.06	0.06	0.02	0.10	0.03	0.01	0.07	0.05	0.01	0.09
GSCB	0.03	0.00	0.06	0.05	0.01	0.10	0.03	0.01	0.08	0.04	0.01	0.09
GSTOT	0.02	0.00	0.05	0.05	0.02	0.09	0.03	0.01	0.07	0.04	0.01	0.08
HM	0.03	0.00	0.06	0.06	0.02	0.11	0.03	0.01	0.07	0.04	0.01	0.09

^a^ALL, all data; RND, herds selected randomly; GSCB, herds selected based on genetic similarity considering common bulls; GSTOT, herds selected based on genetic similarity considering common bulls and common maternal grandsires; HM, herds selected based on harmonic mean of sire’s progeny size

^b^Results for the sub-setting scenarios are expressed as a deviation from ALL, i.e. after subtracting the results of ALL

In Scenario RND, random subsets of FRA herd data were used to provide an easy-to-implement approach for data sub-setting. By chance, some of the 20 subsets could include better-connected herds, which would result in slightly different average coefficients of genetic similarity across the 20 random samples of Scenario RND (see Additional file 1: Table S8). The three subsets that we analysed had the closest coefficients of genetic similarity to those calculated in Scenario ALL with average differences of − 0.008, − 0.0007 and − 0.012, respectively (see Additional file 1: Table S8).

The estimated $r_{g}$, averaged across the three analysed samples of Scenario RND, were lower than those obtained with Scenario ALL (see Additional file 1: Table S4). In particular, $r_{g}$ were slightly lower in Scenario RND for the direct effect (average difference of − 0.02) and lower for the maternal effect (average difference of − 0.04) (Table 7). However, the largest differences in $r_{g}$ between Scenarios RND and ALL were observed for the direct-maternal effect with average differences of − 0.12 and − 0.11 for within-country and between-country $r_{g}$, respectively. On average, the SE of estimated $r_{g}$ were 0.03 greater for the direct effect in Scenario RND than in ALL, and 0.06 greater for the maternal effect (Table 7). The SE were on average 0.03 and 0.05 greater for the direct-maternal within-country and between-country $r_{g}$, respectively.

In Scenario GSCB, $r_{g}$ were estimated based on the top FRA herds that were ranked based on their coefficient of genetic similarity, including connections provided by CB. The coefficients of genetic similarity of selected FRA herds ranged from 0.07 to 0.11. In total, 36% of the FRA herds had a coefficient of genetic similarity lower than 0.001, which indicates a low use of international semen and that their contribution to the estimation of across-country $r_{g}$ is small. The Scenario GSCB resulted, across the selected FRA herds, in an average increase of genetic similarity of 0.15 with other populations compared to Scenario ALL.

Across-country estimated $r_{g}$ from Scenario GSCB were on average lower than from Scenario ALL by 0.02 for the direct and 0.05 for the maternal effect (Table 7). Direct-maternal $r_{g}$ from Scenario GSCB were also on average smaller than from Scenario ALL, by 0.11 within-country and 0.13 between-country. On average, the SE of estimated $r_{g}$ were 0.03 and 0.05 larger for the direct and maternal $r_{g}$ respectively, in Scenario GSCB than in Scenario ALL (Table 7). The SE of the direct-maternal within-country and between-country $r_{g}$ were on average also 0.03 and 0.04 larger in Scenario GSCB than in Scenario ALL.

In Scenario GSTOT, $r_{g}$ were estimated based on the top FRA herds that were ranked based on their coefficient of genetic similarity, including connections provided by both CB and CMGS. The coefficients of genetic similarity of selected FRA herds ranged from 0.08 to 0.12. The coefficient of genetic similarity was lower than 0.001 for many FRA herds (33%). In Scenario GSTOT, selection of the most connected herds resulted, across FRA herds, in a higher coefficient of genetic similarity (considering only CB) with other populations, with an average increase of 0.14 compared to Scenario ALL.

Direct and maternal $r_{g}$ were on average smaller, by 0.02 and 0.05, respectively, in Scenario GSTOT than in Scenario ALL (Table 7), and the largest differences were related to the direct-maternal within-country and between-country $r_{g}$: on average − 0.13 and − 0.12, respectively. On average, the SE of the estimated direct and maternal $r_{g}$ were 0.02 and 0.05 larger in Scenario GSTOT than in Scenario ALL (Table 7) and the SE of the direct-maternal $r_{g}$ were also on average 0.03 and 0.04 larger in Scenario GSTOT than in Scenario ALL, within-country and between-country, respectively.

The aim of Scenario HM was to select FRA herds based on their harmonic mean coefficient (HM). The average HM coefficient of all FRA herds was 965.7 but large variations were observed across herds, with HM coefficients ranging from 0 (small herds with recorded offspring from unknown sires) up to 15,116. For Scenario HM, the 37 selected herds had an average HM coefficient of 8942. Selecting FRA herds on their HM resulted in an average increase of 0.08 of the coefficient of genetic similarity at the population level for FRA compared to Scenario ALL.

Across-country estimated $r_{g}$ from Scenario HM were on average lower than from Scenario ALL by 0.02 for the direct and 0.06 for the maternal effect (Table 7). The direct-maternal $r_{g}$ in Scenario HM were also smaller on average than in Scenario ALL by 0.11 within-country and 0.10 between-country. On average, the SE of the estimated $r_{g}$ were 0.03 and 0.06 larger for the direct and maternal $r_{g}$, respectively (Table 7). Similarly, the SE of the direct-maternal $r_{g}$ from Scenario HM were on average, 0.03 and 0.04 larger than from Scenario ALL, within-country and between-country, respectively.

### Computational requirements

Using all the data, Scenario ALL took 43 days and 23 h to estimate across-country $r_{g}$ (Table 8). The data sub-setting scenarios (RND, GSCB, GSTOT, HM) that were aimed at reducing the number of phenotypes to 0.5 million, decreased the total computing time by 9 to 16 days (Table 8), corresponding to 22% up to 36% of the time required for Scenario ALL. Differences in computational requirements between data sub-setting scenarios can be due to differences in CPU frequency, but also to external factors, such as the server’s load. Considering this, the total computing time was similar across the different data sub-setting scenarios. Random access memory (RAM) requirements were low for all scenarios and ranged from 2.39 gigabytes when using all the data to about 0.5 gigabytes with reduced data (Table 8). Sub-setting scenarios decreased the required time per REML round to ~ 17% of that for Scenario ALL (Table 8). Compared to Scenario ALL, sub-setting the data led to an increase of the number of REML rounds, which ranged from 22% (Scenario RND sample 15) to 43% (Scenario HM).

**Table 8:** Computational requirements across scenarios based on single-core analyses

Scenario^a^	Phenotypic records	Pedigree records	CPU (GHz)^b^	RAM peak usage (GB)^c^	REML rounds (number)	Average time per REML round (min)	Total time (days: hours)^d^
ALL	3,115,598	3,431,742	4.0	2.39	1173	53	43:23
RND sample 15	533,816	706,717	4.0	0.49	1432	11	11:12
RND sample 20	521,077	692,205	3.7	0.48	1543	14	15:21
RND sample 2	556,100	729,778	3.7	0.51	1462	15	16:00
GSCB	506,080	654,841	4.0	0.46	1496	9	9:21
GSTOT	513,969	663,127	4.0	0.45	1530	9	10:06
HM	502,716	649,081	4.0	0.46	1675	9	11:06

^a^ALL, all data; RND, herds selected randomly; GSCB, herds selected based on genetic similarity considering common bulls; GSTOT, herds selected based on genetic similarity considering common bulls and common maternal grandsires; HM, herds selected based on harmonic mean of sire’s progeny size

^b^Central Processing Unit (CPU) frequency

^c^Random access memory (RAM) peak usage

^d^Total elapsed time

## Discussion

In this study, we applied a multi-trait approach to estimate a 16 × 16 across-country $r_{g}$ matrix for Limousin beef cattle international evaluation in a single analysis. Furthermore, we investigated the application of within-country sub-setting strategies to reduce the amount of data required for estimating $r_{g}$. We applied these sub-setting strategies only to the FRA herds, because this is the largest population in the evaluation. Sub-setting the data from the other countries would yield only relatively small reductions in overall data size but could cause loss of valuable information and genetic connections. Our approach could be applied to international beef cattle evaluations of other breeds with population structures similar to that of the Limousin breed, e.g. for country that has a much larger population than the others. An example is the Charolais breed in Interbeef evaluations, with the French population representing more than 90% of the overall data [22, 23].

Hereafter, first we discuss the genetic level of the connections available in the dataset used, then the impact of the sub-setting strategies on the estimated $r_{g}$ and the required computing time. Finally, we describe possible implications of this study in the context of beef cattle international evaluations.

### Genetic connections across-country

Direct connections varied greatly across countries as indicated by the number of CB (Table 2). Most of the CB in this study connected only two populations, but a good proportion of CB connected more than two populations at the same time, most of which were born after 1980 (see Additional file 2: Figure S1). Across-country genetic connections also occurred through CMGS, most of them connecting only two or three populations. However, CMGS that connected more than four populations increasingly appeared as CB (79, 91, 96 and 100% from 5 up to 8 connected populations, respectively). This increased proportion indicates that daughters of popular imported sires are likely to be kept as dams for the next generation and, in turn, will provide grand-offspring’s phenotypes for such sires. In this study, the range of the numbers of CB and CMGS that connected Limousin populations were similar to those previously reported [9, 24, 25], and only a few populations had a small number of connecting CB, but none had missing connections. Limited across-country connections were reported in previous studies both for the Charolais [9, 25] and Simmental [26] breeds. Furthermore, previous studies in dairy cattle breeds, such as Guernsey [27], Ayrshire [5, 18], Brown Swiss and Jersey [5], also reported low across-country connectedness levels, which suggest that our approach may also be beneficial to low-connected dairy breeds. Nevertheless, the number of available connections in the Limousin populations in our study is still small compared to that of dairy breeds such as the Holstein–Friesian [5, 28].

The coefficient of genetic similarity provides a quantification of the number of genetic connections available between two countries. The coefficients of genetic similarity revealed that IRL and FRA are “link-provider” countries, as termed in dairy cattle international evaluations [5, 6, 29]. The inclusion of such link-provider countries, together with the less connected ones, during the estimation of across-country $r_{g}$ may help to overcome the lack of convergence [6], either when including all the countries together in the model or only a group of them, i.e. when applying a country sub-setting strategy.

The sire’s AN_POP coefficient provided further insights on the level of genetic connectedness available in the data. Jorjani et al. [6] showed that selecting sires with a balanced offspring’s distribution may be beneficial to estimate across-country $r_{g}$ in large dairy populations. When applied to international beef evaluations, about 65% of the CB had an AN_POP lower than 2. In addition, all CB with connections in all eight populations were severely penalized for being unbalanced. These results indicate that the majority of the genetic connections are established between two countries and that the number of offspring for CB is unbalanced in beef cattle international evaluations. These unbalanced distributions of sires’ offspring imply that implementing a herd sub-setting strategy for the estimation of across-country $r_{g}$ based on AN_POP was not possible.

### Estimated genetic correlations using all data

The across-country estimated $r_{g}$ and heritabilities obtained in our study are in line with those from previous international beef cattle studies [9, 25]. Estimated direct-maternal within-country $r_{g}$ were negative and ranged from − 0.10 to − 0.33, with the exception of CHE. Using national data, CHE reported a direct-maternal within-country $r_{g}$ of − 0.01 [30]; the positive direct-maternal $r_{g}$ that we obtained for CHE may be related to its sire-by-herd interaction effect (see Additional file 1: Table S1). Berweger et al. [31] also observed that fitting a sire-by-herd interaction effect in the model affected the estimation of direct-maternal $r_{g}$ in Swiss beef cattle data.

In cattle international evaluations, across-country $r_{g}$ determine to what extent the information from participating countries contributes to the estimation of International EBV [32] and can be lower than 1 for different reasons. First, countries may differ in terms of environmental conditions [33], which can lead to genotype-by-environment (i.e. country) interactions [34]. Second, national trait definitions may differ, for instance, depending on the country, AWW being adjusted to a different time period and with different approaches (see Additional file 1: Table S3). Third, differences in national evaluation procedures may exist [35]: an example in beef cattle is the definition of contemporary groups (CG) (see Additional file 1: Table S1).

Submitted AWW were partly modelled differently by each participating country (see Additional file 1: Table S1). Countries adapt their national model to represent in the best way their production system and national evaluations. Thus, effects that explain specific national genetic evaluations should be included in the international evaluation to account for possible non-genetic sources of variation of the submitted data [35]. Examples of specific national sources of variation are access to alpine grazing, seasonality effects and small CG modelled as random [36]. In international evaluations, both in dairy and beef cattle, it is common practice to have different effects fitted at the national level between countries. In dairy cattle international evaluations, countries correct for their national fixed and random effects before submitting sires’ de-regressed proofs [35, 37]. In Interbeef, since phenotypes are shared across countries, the correction for fixed and random national effects is done in one step at the international level with the AMACI model by modelling each country, and its associated national model, as a different trait [1, 38].

Large SE of across-country estimated $r_{g}$ (Table 6) are common in studies concerning international evaluations, both in beef [1, 25] and dairy cattle [32, 39]. Large SE may be due to a lack of direct connections across-country that are established through CB, and to the heterogeneous data structure between different countries for the same trait [25, 32]. Although in our dataset, there was a medium level of connectedness, in terms of coefficient of genetic similarity, and no population had missing genetic links (i.e. CB = 0), the large SE obtained underline and support the need to increase genetic connections across-country in beef cattle populations.

Estimated variance components depend, to some extent, on the information content of the data. For instance, it is important that the performance of an animal can be compared to contemporaries within herd. In international beef cattle evaluations, the minimum size of the CG is defined by each participating country (Table 1). Two populations (CZE and DFS) reported a minimum size of CG of 1. However, in Scenario ALL, the number of animals belonging to a CG of size 1 was limited to only 0.1% of the total phenotypes. We tested the hypothesis that animals belonging to a CG of size 1 had no impact on the results of this study: the maximum difference in $r_{g}$ with ALL was 0.009, for a direct-maternal between-country $r_{g}$ (results not shown).

Removing old data from the process to estimate variance components is an alternative straightforward approach to reduce the amount of data used. We investigated the effect of removing old disconnected Limousin beef cattle data on the across-country estimated $r_{g}$. Since most of the across-country genetic connections, established through CB that connected more than two populations, were initiated after 1980 (see Additional file 2: Figure S1), we chose this year as a threshold for the recorded year of birth of an animal. The retained data included 3,019,527 phenotypes, with a pruned pedigree of 3,345,349 animals. Removing old disconnected data had a negligible impact on the estimated $r_{g}$ compared to all data in Scenario ALL: average differences of 0.00 for direct and maternal $r_{g}$, and − 0.01 for direct-maternal within-country and between-country $r_{g}$ (results not shown). This limited impact on across-country estimated $r_{g}$ is in agreement with the findings of Jorjani [28] in dairy cattle international evaluations.

### Impact of reducing data

In this study, we shifted the focus from the selection of the most connected sires, which is typical of international dairy cattle evaluations [6], to the selection of the management unit, i.e. the most connected herds. Selection of entire herds better accounts for beef cattle data structure and allows retaining all within-herd CG information for those dams and sires with multiple recorded offspring. In turn, selecting herds may be beneficial for the estimation of maternal genetic effects. The coefficient of genetic similarity applied at the herd level as in Scenarios GSCB and GSTOT do not account for the herd size. Nevertheless, we observed a linear relationship between the coefficient of genetic similarity of herd and the herd size. A possible explanation for this positive relationship is that small herds may be more inclined to use natural service bulls, as opposed to large herds, in which the more frequent use of artificial insemination leads to a higher use of international sires’ semen. Furthermore, herd coefficients of genetic similarity revealed a large proportion (more than 30%) of FRA herds disconnected from other populations, and in all non-random scenarios (GSCB, GSTOT and HM), larger herds, compared to Scenario ALL, were selected: average number of records per herd of 406.5, 1588.6, 1708.2 and 2742.9 for Scenarios ALL, GSCB, GSTOT and HM, respectively. Likewise, compared to Scenario ALL, larger CG were selected in non-random scenarios: average number of records per CG of 16.2, 20.9, 21.7 and 30.6 for Scenarios ALL, GSCB, GSTOT and HM, respectively.

Genetic similarity computed at the FRA population level increased in non-random scenarios compared to Scenario ALL, even if some CB between FRA and other populations were removed due to the herd selection process. Nevertheless, estimated $r_{g}$ are similar across sub-setting scenarios. All sub-setting scenarios resulted in smaller across-country $r_{g}$, which ranged from an average of 0.02 for the direct $r_{g}$ up to 0.13 for the direct-maternal within-country $r_{g}$. The SE of estimated $r_{g}$ always increased when reduced data were used, regardless of the scenario considered, and ranged from an average increment of 0.02 up to 0.06. The average difference in estimated $r_{g}$ between Scenarios GSCB and GSTOT was 0.00 (regardless of the effect considered), which indicated that there was no benefit in including information from CMGS in the coefficient of genetic similarity. Results from non-random scenarios suggest that given a certain level of genetic connections, as in Scenario GSCB, there is no additional benefit in including information related to CMGS, as in Scenario GSTOT, or to the CB’s balanced offspring distribution, as in Scenario HM.

To further understand the impact of reducing FRA data on across-country estimated $r_{g}$, we performed an additional scenario (NO_FRA), i.e. by using the same dataset as in Scenario ALL, but the FRA population was excluded from the estimation process. The results showed that the inclusion of FRA data helps to estimate the national genetic variances of other populations (see Additional file 2: Figures S2 and S3). For example, smaller maternal genetic variance and larger maternal permanent environmental variance were estimated for IRL in NO_FRA than in Scenario ALL, leading to a lower maternal heritability for IRL in NO_FRA than in Scenario ALL. On the other hand, for the GBR population, the exclusion of FRA data resulted in a larger estimated maternal genetic variance than in Scenario ALL. In addition, compared with Scenario ALL, reducing or removing FRA data resulted in a larger estimated maternal permanent environmental variance for ESP (see Additional file 2: Figure S2). Thus, it seems that differences in across-country estimated $r_{g}$ across scenarios are related to the amount of FRA data used in the estimation process, rather than to how the subset of FRA data was chosen. Thus, we foresee that the application of other methods for the selection of connected herds, even if they are more sophisticated such as the CD methodology [40], would not result in across-country estimated $r_{g}$ closer to those obtained when using of all data. After all, differences in estimated $r_{g}$ between Scenario ALL and other scenarios were small.

While Scenario RND reduced the number of FRA herds randomly, Scenarios GSCB, GSTOT and HM reduced the number of observations by selecting the best-connected herds. Therefore, selected data are not “missing-at-random” [41, 42]. Bayesian algorithms have been suggested to be better suited for dealing with such non-missing-at-random data structures compared to REML algorithms [41]. Nevertheless, in our study, scenarios with non-random sub-setting showed small differences in estimated $r_{g}$ compared to those of Scenario RND.

The total required computing time was similar for all sub-setting scenarios, with the computing time per REML round being directly proportional to the data reduction applied. Indeed, by reducing data to ~ 16% of the complete dataset (from 3.1 to ~ 0.5 million phenotypes), the required computing time per REML round was reduced to ~ 17% (from 53 min in Scenario ALL to 9 min in non-random scenarios). However, the larger required number of REML rounds in sub-setting scenarios resulted in the total computing time not being proportional to the data reduction applied (Table 8). A larger number of REML rounds may indicate a more difficult estimation of the parameters during VCE as suggested by Jorjani et al. [6]. In this study, selecting herds based on the coefficient of genetic similarity as in Scenarios GSCB and GSTOT, required a smaller number of REML rounds than in Scenario HM, which indicates that herds selected based on genetic similarity could result in an easier estimation of genetic parameters. Although Scenario RND subsets resulted in a similar number of REML rounds compared to Scenarios GSCB and GSTOT, the required computing time was longer due to the larger number of records analysed.

### Implications

Estimated within-country genetic and environmental variances may differ from those reported by member countries, both when estimated using all international data (ALL Scenario) and subsets of data (RND, GSCB, GSTOT, HM Scenarios) (see Additional file 2: Figures S2 and S3). However, national reported variances are considered more accurate than those obtained from international data, since countries can use a more representative national dataset for VCE, e.g. when not all the data are submitted for international evaluations. Thus, in Interbeef evaluations, changes in across-country $r_{g}$ are of primary interest, whereas within-country estimated variances are replaced with those reported by member countries. Indeed, it is common practice to use the reported national variances together with the estimated across-country $r_{g}$ to build the across-country genetic covariance matrix from which IEBV are estimated.

The multi-variate approach proposed in this study is preferable over the current-in-use bi-variate approach for Interbeef evaluations [9]: matrices obtained in different scenarios were positive definite and bending procedures were avoided. This was enabled by the MC EM REML algorithm that allowed the estimation of all 120 across-country $r_{g}$ at the same time. In comparison, when using the bi-variate method with a similar dataset as in this study, many estimated across-country $r_{g}$ did not converge, for example, 8 and 17 out of the 28 $r_{g}$, for the direct and maternal effects, respectively (Pabiou, personal communication). As a result, across-country $r_{g}$ are usually set to an arbitrary default value. The use of such arbitrary values may be questionable, especially considering the possible impact on IEBV and, in turn, on establishing future genetic connections across populations. Moreover, under the current bi-variate approach, all 56 direct-maternal between-country $r_{g}$ are not estimated but are assumed to be 0, which may not be realistic. For instance, using all data, direct-maternal between-country estimated $r_{g}$ were all negative for FRA (Table 6), while for CHE they were almost all positive.

## Conclusions

The MC EM REML algorithm allowed the simultaneous estimation of all across-country $r_{g}$ using a multi-trait animal model. Reducing the data mainly affected the estimates of direct-maternal within-country and between-country $r_{g}$, but had a small impact on the estimated direct and maternal across-country $r_{g}$. Estimated $r_{g}$ were very similar across sub-setting strategies, which means that the way the subset of FRA data was chosen hardly affected the values of $r_{g}$. The standard errors of estimated $r_{g}$ increased when reduced data were used. Using the data sub-setting strategies reduced the amount of data used to about 16% of the complete dataset and the required computing time decreased to 22% of that required when using all data.

## Supplementary information

**Additional file 1: Table S1.** List of fixed, random environmental and covariate environmental effects in the national model per each population. **Table S2.** Number of phenotypes (N), minimum, phenotypic mean, maximum and phenotypic standard deviation ($\sigma_{P}$) of males and females per population. **Table S3.** Age of adjustment, recording time frame, editing and adjusting criteria for weaning weight records in each population as reported in Interbeef national Genetic Evaluation forms in 2018 [30]. **Table S4.** Differences in estimated genetic correlations for direct and maternal genetic effects (below diagonal) and their standard errors (above diagonal) between RND and ALL. **Table S5.** Differences in estimated genetic correlations for direct and maternal genetic effects (below diagonal) and their standard errors (above diagonal) between GSCB and ALL. **Table S6.** Differences in estimated genetic correlations for direct and maternal genetic effects (below diagonal) and their standard errors (above diagonal) between GSTOT and ALL. **Table S7.** Differences in estimated genetic correlations for direct and maternal genetic effects (below diagonal) and their standard errors (above diagonal) between HM and ALL. **Table S8.** Average differences in GS between each random subset of FRA-herds of RND and ALL.

**Additional file 2: Figure S1.** Adjusted Number of Populations (AN_POP) for all common bulls in function of their year of birth and country of first registration. **Figure S2.** Estimated (co)variance components per population across different scenarios. **Figure S3.** Estimated random contemporary group and sire by herd interaction variances across different scenarios.

## Data Availability

All information supporting the results are included in this article and its additional files. The data that support the findings of this study are available at Interbeef. Restrictions apply to the availability of these data, which were used under license for the current study.
