# Supplementary material for: Impact of sub-setting the data of the main Limousin beef cattle population on the estimates of across-country genetic correlations
Source: Genet Sel Evol. 2020 Jun 23;52:32. doi: 10.1186/s12711-020-00551-9 (PMC7310393; doi:10.1186/s12711-020-00551-9)
Supplement: Supplementary file 1 — Additional file 1: Table S1. List of fixed, random environmental and covariate environmental effects in the national model per each population. Table S2. Number of phenotypes (N), minimum, phenotypic mean, maximum and phenotypic standard deviation ($\sigma_{P}$σP) of males and females per population. Table S3. Age of adjustment, recording time frame, editing and adjusting criteria for weaning weight records in each population as reported in Interbeef national Genetic Evaluation forms in 2018 [30]. Table S4. Differences in estimated genetic correlations for direct and maternal genetic effects (below diagonal) and their standard errors (above diagonal) between RND and ALL. Table S5. Differences in estimated genetic correlations for direct and maternal genetic effects (below diagonal) and their standard errors (above diagonal) between GSCB and ALL. Table S6. Differences in estimated genetic correlations for direct and maternal genetic effects (below diagonal) and their standard errors (above diagonal) between GSTOT and ALL. Table S7. Differences in estimated genetic correlations for direct and maternal genetic effects (below diagonal) and their standard errors (above diagonal) between HM and ALL. Table S8. Average differences in GS between each random subset of FRA-herds of RND and ALL. [file 12711_2020_551_MOESM1_ESM.docx]

# Additional file 1: Tables S1-S8

Table S1. List^1^ of fixed, random environmental and covariate environmental effects in the national model per each population.

| POP^2^ | Fixed | | | | | | | Random | | | Covariates | |
| --- | --- | --- | --- | --- | --- | --- | --- | --- | --- | --- | --- | --- |
| CZE |  | asextwin | aaca | year |  |  |  | PE | HYS |  |  |  |
| DFS | HYS | asex | aaca | seas | twin |  |  | PE |  |  |  |  |
| ESP | herd_birth | asex | aaca |  |  |  |  | PE |  |  |  |  |
| GBR | HYS_mgt | asex |  | month | twin | fostered | wdam_brd | PE |  |  | agedam | agedam2 |
| IRL | HYS | asex | pariagedam |  |  |  |  | PE |  |  | agedam2 | aawg |
| FRA | HY-asex-mgt |  | pariaaca | seas |  |  | individual | PE |  |  |  |  |
| DEU |  | asex | parity | month | twin |  |  |  | HY |  |  |  |
| CHE |  | asex |  | yearmonth |  |  | alpine | PE | HY | SireHerd | agedam | agedam2 |

^1^ *aaca* = age at calving; *aawg* = age at weighting; *agedam* = age of the dam; *agedam*2 = age of the dam fitted as quadratic effect; *alpine* = access to alpine grazing for calves; *asex* = sex of the animal; *asextwin* = interaction between *asex* and *twin*; *fostered* = foster code; *herd_birth* = contemporary group defined based on the herd and birth date; *HY* = Herd-Year; *HY-asex-mgt* = contemporary group defined based on *HY*, *asex* and management group defined as calf‐dam couple; *HYS* = Herd-Year-Season; *HYS_mgt* = contemporary group defined by herd, management group and date of birth; *individual* = individual situation, e.g. preferential treatment; *month* = month of birth; *pari* = parity; *pariaaca* = interaction between *pari* and *aaca* effects; *pariagedam* = interaction between *pari* and *agedam*; *PE* = maternal permanent environmental effect; *seas* = season; *SireHerd* = interaction between sire and herd; *twin* = twinning; *wdam_brd* = breed of the weaning dam; *year* = year of birth; *yearmonth* = interaction between *year* and *month*.

^2^ Population: CZE = Czech Republic, DFS = Denmark, Finland and Sweden, ESP = Spain, GBR = Great Britain, IRL = Ireland, FRA = France, DEU = Germany, CHE = Switzerland.

Table S2. Number of phenotypes (N), minimum, phenotypic mean, maximum and phenotypic standard deviation ($\sigma_{P}$) of males and females per population.

|  | Males | | | | | Females | | | | |
| --- | --- | --- | --- | --- | --- | --- | --- | --- | --- | --- |
| POP^1^ | **N** | **Min** | **Mean** | **Max** | $\boldsymbol{\sigma}_{\boldsymbol{P}}$ | **N** | **Min** | **Mean** | **Max** | $\boldsymbol{\sigma}_{\boldsymbol{P}}$ |
| CZE | 5,119 | 176 | 292.84 | 407 | 36.71 | 5,381 | 155 | 262.07 | 366 | 33.64 |
| DFS | 45,191 | 111 | 239.60 | 368 | 41.55 | 45,265 | 106 | 212.22 | 316 | 33.99 |
| ESP | 16,416 | 147 | 268.01 | 376 | 43.70 | 16,736 | 147 | 245.94 | 361 | 38.14 |
| GBR | 62,980 | 158 | 288.85 | 419 | 42.57 | 64,860 | 145 | 254.19 | 363 | 35.44 |
| IRL | 11,370 | 120 | 286.24 | 454 | 55.20 | 9,239 | 118 | 257.51 | 397 | 46.89 |
| FRA | 1,324,990 | 154 | 277.34 | 400 | 40.53 | 1,389,378 | 148 | 250.07 | 352 | 33.41 |
| DEU | 43,725 | 135 | 266.83 | 398 | 43.01 | 44,903 | 126 | 239.74 | 353 | 36.99 |
| CHE | 15,323 | 112 | 232.12 | 351 | 38.87 | 14,722 | 106 | 207.86 | 310 | 32.92 |

^1^ Population: CZE = Czech Republic, DFS = Denmark, Finland and Sweden, ESP = Spain, GBR = Great Britain, IRL = Ireland, FRA = France, DEU = Germany, CHE = Switzerland.

Table S3. Age of adjustment, recording time frame, editing and adjusting criteria for weaning weight records in each population as reported in Interbeef national Genetic Evaluation forms in 2018 [30].

| POP^a^ | Adjustment (days) | Recording time  (days) | Editing criteria | Adjustment criteria |
| --- | --- | --- | --- | --- |
| CZE | 210 | 171-290 | Incomplete data removed.  Data over 3 standard deviations from breed-sex mean are removed. | - |
| DFS | 200 | 140-260 (DNK^a^)  150-250 (FIN^a^)  125-275 (SWE^a^) | Incomplete data removed. | $200 \cdot\frac{{WW}^{d}-{BW}^{b}}{Weaning age-Birth age}$ |
| ESP | 210 | BW^b^ – 300 days | Daily weight gain limit: 0.0 - 2.5 Kg.  At least 2 weights (one after 60 days). | Individual linear regression |
| GBR | 200 | 170-300 | Max weight: 500 kg. | Interpolation of the two nearest weights before and after 200 days |
| IRL | 200 | 150-300 | - | Average of recorded weights |
| FRA | 210 | At least 2 weights recorded within 300 days; each at maximum 2 months apart from the 210 days weight. | If 210 days AWW^c^ is not possible to be calculated, a 120-days weight is considered. | Individual intra-extrapolation |
| DEU | 200 | 90-280 | Incomplete data removed. | - |
| CHE | 200 | 90-320 | Daily weight gain limit: 0.3 - 2.5 Kg.  Animals without WW^d^ and incomplete information are removed. | $200 \cdot\frac{W{WW}^{d}-{BW}^{b}}{Weaning age}$ |

^a^ Population: CZE = Czech Republic, DFS = Denmark, Finland and Sweden, ESP = Spain, GBR = Great Britain, IRL = Ireland, FRA = France, DEU = Germany, CHE = Switzerland, DNK = Denmark, FIN = Finland, SWE = Sweden. ^b^ BW = Birth Weight. ^c^ AWW = Age-adjusted Weaning Weight. ^d^ WW = weaning weight.

Table S4. Differences in estimated genetic correlations for direct and maternal genetic effects (below diagonal) and their standard errors (above diagonal) between RND and ALL^1,2^.

|  |  | DIRECT | | | | | | | | MATERNAL | | | | | | | |
| --- | --- | --- | --- | --- | --- | --- | --- | --- | --- | --- | --- | --- | --- | --- | --- | --- | --- |
|  |  | CZE | DFS | ESP | GBR | IRL | FRA | DEU | CHE | CZE | DFS | ESP | GBR | IRL | FRA | DEU | CHE |
| DIRECT | CZE |  | 0.02 | **0.05** | 0.03 | 0.02 | 0.03 | 0.02 | 0.03 | 0.04 | 0.04 | **0.09** | **0.07** | 0.05 | **0.05** | 0.03 | **0.06** |
|  | DFS | -0.02 |  | 0.03 | 0.02 | 0.02 | 0.03 | 0.01 | 0.04 | 0.03 | 0.01 | **0.06** | **0.05** | 0.03 | 0.05 | 0.02 | 0.03 |
|  | ESP | -0.03 | -0.03 |  | 0.04 | 0.04 | 0.04 | 0.03 | **0.06** | **0.06** | 0.04 | **0.07** | **0.06** | 0.05 | **0.07** | 0.03 | **0.07** |
|  | GBR | -0.04 | -0.02 | -0.01 |  | 0.02 | 0.04 | 0.02 | 0.03 | 0.05 | 0.03 | **0.05** | 0.02 | 0.03 | **0.06** | 0.03 | **0.06** |
|  | IRL | -0.02 | -0.02 | -0.02 | -0.01 |  | 0.03 | 0.02 | 0.03 | 0.04 | 0.03 | 0.04 | 0.03 | 0.02 | **0.05** | 0.03 | **0.05** |
|  | FRA | -0.03 | -0.01 | -0.02 | -0.01 | -0.02 |  | 0.04 | 0.04 | 0.05 | 0.03 | **0.08** | **0.06** | 0.05 | **0.07** | 0.04 | **0.06** |
|  | DEU | -0.02 | -0.01 | -0.02 | -0.02 | -0.03 | -0.01 |  | 0.04 | 0.03 | 0.01 | **0.05** | 0.05 | 0.03 | 0.05 | 0.01 | 0.04 |
|  | CHE | -0.02 | -0.02 | -0.02 | -0.03 | -0.03 | -0.03 | -0.03 |  | 0.03 | 0.05 | **0.06** | **0.06** | 0.04 | **0.06** | 0.03 | 0.02 |
| MATERNAL | CZE | **-0.11** | **-0.12** | **-0.10** | **-0.10** | **-0.09** | **-0.10** | **-0.11** | **-0.11** |  | 0.04 | **0.10** | **0.07** | 0.05 | **0.06** | 0.03 | **0.06** |
|  | DFS | **-0.14** | **-0.17** | **-0.14** | **-0.14** | **-0.12** | **-0.13** | **-0.16** | **-0.14** | -0.05 |  | **0.07** | 0.05 | 0.04 | **0.07** | 0.02 | **0.05** |
|  | ESP | **-0.12** | **-0.14** | **-0.16** | **-0.14** | **-0.12** | **-0.12** | **-0.14** | **-0.14** | **-0.05** | -0.04 |  | **0.07** | **0.07** | **0.10** | 0.03 | 0.05 |
|  | GBR | **-0.09** | **-0.12** | **-0.12** | **-0.14** | **-0.10** | **-0.12** | **-0.12** | **-0.12** | -0.03 | -0.05 | **-0.06** |  | 0.04 | **0.08** | 0.04 | **0.10** |
|  | IRL | **-0.12** | **-0.13** | **-0.13** | **-0.13** | **-0.12** | **-0.11** | **-0.12** | **-0.11** | **-0.05** | -0.05 | -0.03 | **-0.05** |  | **0.05** | 0.02 | **0.08** |
|  | FRA | **-0.11** | **-0.14** | **-0.12** | **-0.13** | **-0.11** | **-0.14** | **-0.13** | **-0.11** | -0.03 | -0.04 | -0.05 | -0.02 | -0.03 |  | **0.07** | **0.09** |
|  | DEU | **-0.07** | **-0.08** | **-0.08** | **-0.08** | **-0.06** | **-0.06** | **-0.10** | **-0.07** | -0.03 | -0.03 | -0.03 | -0.04 | -0.03 | -0.04 |  | 0.04 |
|  | CHE | **-0.07** | **-0.09** | **-0.09** | **-0.09** | **-0.08** | **-0.08** | **-0.10** | -0.04 | -0.05 | **-0.06** | **-0.06** | **-0.07** | **-0.06** | -0.04 | -0.04 |  |

^1^ Differences greater than 0.05 are reported in bold. ^2^ Population: CZE = Czech Republic, DFS = Denmark, Finland and Sweden, ESP = Spain, GBR = Great Britain, IRL = Ireland, FRA = France, DEU = Germany, CHE = Switzerland. RND = herds selected randomly, ALL = all data.

Table S5. Differences in estimated genetic correlations for direct and maternal genetic effects (below diagonal) and their standard errors (above diagonal) between GSCB and ALL^1,2^.

|  |  | DIRECT | | | | | | | | MATERNAL | | | | | | | |
| --- | --- | --- | --- | --- | --- | --- | --- | --- | --- | --- | --- | --- | --- | --- | --- | --- | --- |
|  |  | CZE | DFS | ESP | GBR | IRL | FRA | DEU | CHE | CZE | DFS | ESP | GBR | IRL | FRA | DEU | CHE |
| DIRECT | CZE |  | 0.01 | **0.05** | 0.04 | 0.02 | 0.04 | 0.02 | 0.01 | 0.03 | 0.03 | **0.09** | **0.06** | 0.02 | 0.04 | 0.03 | 0.03 |
|  | DFS | -0.01 |  | 0.03 | 0.01 | 0.02 | 0.04 | 0.00 | 0.04 | 0.03 | 0.01 | **0.06** | 0.04 | 0.03 | 0.04 | 0.02 | 0.03 |
|  | ESP | -0.03 | -0.02 |  | 0.03 | 0.02 | 0.04 | 0.02 | **0.06** | 0.05 | 0.02 | **0.06** | **0.06** | 0.03 | **0.07** | 0.02 | 0.04 |
|  | GBR | -0.03 | -0.02 | -0.01 |  | 0.01 | 0.04 | 0.01 | 0.02 | 0.05 | 0.02 | **0.06** | 0.01 | 0.02 | **0.05** | 0.03 | 0.04 |
|  | IRL | -0.01 | -0.01 | -0.01 | -0.01 |  | 0.04 | 0.02 | 0.00 | **0.05** | 0.02 | 0.04 | 0.03 | 0.02 | 0.04 | 0.04 | **0.06** |
|  | FRA | -0.02 | 0.00 | -0.01 | -0.01 | -0.01 |  | 0.04 | 0.04 | **0.05** | 0.04 | **0.07** | **0.05** | 0.05 | **0.08** | 0.03 | **0.05** |
|  | DEU | -0.02 | -0.01 | -0.02 | -0.02 | -0.02 | -0.01 |  | 0.02 | 0.03 | 0.01 | **0.06** | 0.03 | 0.03 | 0.05 | 0.01 | 0.04 |
|  | CHE | -0.02 | -0.02 | -0.02 | -0.02 | -0.02 | -0.01 | -0.02 |  | 0.02 | 0.02 | **0.06** | **0.05** | 0.02 | 0.04 | 0.01 | 0.02 |
| MATERNAL | CZE | **-0.12** | **-0.12** | **-0.11** | **-0.10** | **-0.09** | **-0.09** | **-0.12** | **-0.12** |  | 0.01 | **0.07** | **0.06** | 0.05 | **0.05** | 0.02 | 0.04 |
|  | DFS | **-0.14** | **-0.17** | **-0.14** | **-0.15** | **-0.12** | **-0.13** | **-0.17** | **-0.15** | **-0.06** |  | 0.05 | 0.05 | 0.05 | **0.06** | 0.02 | 0.05 |
|  | ESP | **-0.13** | **-0.14** | **-0.17** | **-0.15** | **-0.12** | **-0.11** | **-0.15** | **-0.15** | **-0.06** | -0.05 |  | **0.07** | **0.07** | **0.10** | 0.04 | 0.04 |
|  | GBR | **-0.09** | **-0.13** | **-0.13** | **-0.15** | **-0.10** | **-0.10** | **-0.13** | **-0.13** | -0.04 | **-0.06** | **-0.07** |  | 0.03 | **0.07** | 0.03 | **0.09** |
|  | IRL | **-0.12** | **-0.13** | **-0.14** | **-0.14** | **-0.13** | **-0.10** | **-0.13** | **-0.12** | **-0.07** | **-0.05** | -0.04 | **-0.07** |  | **0.05** | 0.04 | **0.09** |
|  | FRA | **-0.12** | **-0.15** | **-0.14** | **-0.15** | **-0.12** | **-0.13** | **-0.15** | **-0.13** | -0.03 | -0.05 | **-0.05** | -0.03 | -0.04 |  | **0.07** | **0.08** |
|  | DEU | **-0.06** | **-0.07** | **-0.07** | **-0.07** | -0.05 | -0.03 | **-0.10** | **-0.06** | -0.04 | -0.04 | -0.04 | -0.04 | -0.04 | -0.05 |  | 0.03 |
|  | CHE | **-0.07** | **-0.09** | **-0.09** | **-0.09** | **-0.07** | **-0.06** | **-0.10** | -0.04 | **-0.06** | **-0.07** | **-0.07** | **-0.08** | **-0.08** | **-0.06** | **-0.05** |  |

^1^ Differences greater than 0.05 are reported in bold. ^2^ Population: CZE = Czech Republic, DFS = Denmark, Finland and Sweden, ESP = Spain, GBR = Great Britain, IRL = Ireland, FRA = France, DEU = Germany, CHE = Switzerland. GSCB = herds selected based on genetic similarity considering common bulls, ALL = all data.

Table S6. Differences in estimated genetic correlations for direct and maternal genetic effects (below diagonal) and their standard errors (above diagonal) between GSTOT and ALL^1,2^.

|  |  | DIRECT | | | | | | | | MATERNAL | | | | | | | |
| --- | --- | --- | --- | --- | --- | --- | --- | --- | --- | --- | --- | --- | --- | --- | --- | --- | --- |
|  |  | CZE | DFS | ESP | GBR | IRL | FRA | DEU | CHE | CZE | DFS | ESP | GBR | IRL | FRA | DEU | CHE |
| DIRECT | CZE |  | 0.01 | 0.04 | 0.03 | 0.02 | 0.03 | 0.01 | 0.01 | 0.04 | 0.05 | **0.08** | **0.07** | 0.05 | 0.04 | 0.02 | **0.05** |
|  | DFS | -0.01 |  | 0.02 | 0.02 | 0.02 | 0.03 | 0.00 | 0.03 | 0.02 | 0.01 | 0.05 | **0.06** | 0.03 | 0.03 | 0.01 | 0.04 |
|  | ESP | -0.03 | -0.02 |  | 0.03 | 0.03 | 0.03 | 0.02 | **0.05** | 0.04 | 0.02 | **0.07** | 0.04 | **0.05** | **0.06** | 0.01 | 0.03 |
|  | GBR | -0.03 | -0.02 | -0.01 |  | 0.01 | 0.04 | 0.01 | 0.02 | 0.03 | 0.02 | 0.04 | 0.02 | 0.02 | **0.05** | 0.01 | **0.06** |
|  | IRL | -0.01 | -0.01 | -0.01 | -0.01 |  | 0.04 | 0.03 | 0.02 | 0.04 | 0.03 | 0.04 | 0.03 | 0.03 | 0.05 | 0.02 | **0.05** |
|  | FRA | -0.02 | 0.00 | -0.01 | -0.01 | -0.01 |  | 0.03 | 0.03 | **0.05** | 0.04 | **0.08** | **0.07** | **0.05** | **0.07** | 0.03 | **0.06** |
|  | DEU | -0.02 | -0.01 | -0.02 | -0.02 | -0.02 | -0.01 |  | 0.03 | 0.04 | 0.02 | 0.05 | 0.03 | 0.04 | 0.04 | 0.01 | 0.04 |
|  | CHE | -0.02 | -0.02 | -0.02 | -0.02 | -0.03 | -0.01 | -0.03 |  | 0.03 | 0.04 | **0.07** | **0.06** | 0.03 | 0.03 | 0.01 | 0.02 |
| MATERNAL | CZE | **-0.12** | **-0.12** | **-0.11** | **-0.11** | **-0.10** | **-0.09** | **-0.12** | **-0.12** |  | 0.04 | **0.06** | **0.06** | 0.03 | **0.07** | 0.03 | **0.06** |
|  | DFS | **-0.14** | **-0.18** | **-0.14** | **-0.15** | **-0.12** | **-0.13** | **-0.17** | **-0.15** | **-0.06** |  | 0.04 | 0.04 | 0.04 | **0.06** | 0.02 | 0.05 |
|  | ESP | **-0.13** | **-0.14** | **-0.17** | **-0.15** | **-0.12** | **-0.11** | **-0.16** | **-0.15** | **-0.06** | -0.05 |  | **0.07** | **0.07** | **0.09** | 0.04 | 0.02 |
|  | GBR | **-0.09** | **-0.13** | **-0.13** | **-0.15** | **-0.10** | **-0.11** | **-0.13** | **-0.13** | -0.04 | **-0.06** | **-0.07** |  | 0.04 | **0.07** | 0.03 | **0.08** |
|  | IRL | **-0.12** | **-0.14** | **-0.14** | **-0.14** | **-0.13** | **-0.11** | **-0.13** | **-0.12** | **-0.07** | **-0.05** | -0.04 | **-0.07** |  | **0.06** | 0.03 | **0.08** |
|  | FRA | **-0.13** | **-0.16** | **-0.14** | **-0.15** | **-0.13** | **-0.14** | **-0.15** | **-0.13** | -0.03 | -0.05 | **-0.05** | -0.03 | -0.04 |  | **0.06** | **0.08** |
|  | DEU | **-0.06** | **-0.07** | **-0.07** | **-0.07** | -0.05 | -0.04 | **-0.10** | **-0.06** | -0.04 | -0.04 | -0.04 | -0.04 | -0.04 | -0.05 |  | 0.03 |
|  | CHE | **-0.07** | **-0.09** | **-0.09** | **-0.09** | **-0.08** | **-0.07** | **-0.10** | -0.05 | **-0.06** | **-0.07** | **-0.07** | **-0.08** | **-0.08** | **-0.06** | **-0.05** |  |

^1^ Differences greater than 0.05 are reported in bold. ^2^ Population: CZE = Czech Republic, DFS = Denmark, Finland and Sweden, ESP = Spain, GBR = Great Britain, IRL = Ireland, FRA = France, DEU = Germany, CHE = Switzerland. GSTOT = herds selected based on genetic similarity considering common bulls and common maternal grandsires, ALL = all data.

Table S7. Differences in estimated genetic correlations for direct and maternal genetic effects (below diagonal) and their standard errors (above diagonal) between HM and ALL^1,2^.

|  |  | DIRECT | | | | | | | | MATERNAL | | | | | | | |
| --- | --- | --- | --- | --- | --- | --- | --- | --- | --- | --- | --- | --- | --- | --- | --- | --- | --- |
|  |  | CZE | DFS | ESP | GBR | IRL | FRA | DEU | CHE | CZE | DFS | ESP | GBR | IRL | FRA | DEU | CHE |
| DIRECT | CZE |  | 0.00 | 0.05 | 0.03 | 0.03 | 0.02 | 0.01 | 0.02 | 0.03 | 0.04 | **0.09** | 0.04 | 0.04 | 0.04 | 0.03 | 0.05 |
|  | DFS | -0.02 |  | 0.03 | 0.02 | 0.03 | 0.03 | 0.01 | 0.05 | 0.04 | 0.02 | **0.06** | 0.04 | 0.04 | 0.03 | 0.02 | 0.02 |
|  | ESP | -0.03 | -0.03 |  | 0.03 | **0.06** | 0.03 | 0.02 | **0.06** | 0.05 | 0.02 | **0.07** | **0.06** | 0.04 | **0.06** | 0.02 | 0.04 |
|  | GBR | -0.04 | -0.02 | -0.01 |  | 0.02 | 0.04 | 0.02 | 0.02 | **0.05** | 0.02 | **0.06** | 0.02 | 0.02 | **0.06** | 0.02 | **0.06** |
|  | IRL | -0.02 | -0.02 | -0.02 | -0.01 |  | 0.04 | 0.01 | 0.02 | 0.05 | 0.02 | **0.07** | 0.04 | 0.04 | **0.05** | 0.03 | **0.06** |
|  | FRA | -0.03 | 0.00 | -0.02 | -0.01 | -0.02 |  | 0.04 | 0.04 | **0.05** | 0.04 | **0.08** | 0.05 | 0.05 | **0.07** | 0.04 | **0.06** |
|  | DEU | -0.03 | -0.01 | -0.02 | -0.02 | -0.03 | -0.01 |  | 0.04 | 0.03 | 0.01 | 0.05 | 0.04 | 0.03 | 0.04 | 0.01 | 0.03 |
|  | CHE | -0.02 | -0.02 | -0.02 | -0.03 | -0.03 | -0.02 | -0.03 |  | 0.04 | 0.03 | **0.06** | **0.06** | 0.04 | **0.06** | 0.03 | 0.01 |
| MATERNAL | CZE | **-0.10** | **-0.09** | **-0.08** | **-0.08** | **-0.07** | **-0.05** | **-0.09** | **-0.09** |  | 0.05 | **0.07** | **0.06** | 0.05 | **0.07** | 0.03 | **0.06** |
|  | DFS | **-0.14** | **-0.17** | **-0.14** | **-0.15** | **-0.12** | **-0.12** | **-0.17** | **-0.15** | **-0.06** |  | **0.06** | 0.04 | 0.05 | **0.06** | 0.02 | 0.04 |
|  | ESP | **-0.12** | **-0.13** | **-0.17** | **-0.14** | **-0.12** | **-0.09** | **-0.14** | **-0.14** | **-0.07** | **-0.05** |  | **0.08** | **0.08** | **0.11** | 0.05 | 0.04 |
|  | GBR | **-0.07** | **-0.11** | **-0.11** | **-0.13** | **-0.09** | **-0.08** | **-0.11** | **-0.11** | -0.04 | **-0.06** | **-0.07** |  | 0.03 | **0.07** | 0.04 | **0.09** |
|  | IRL | **-0.11** | **-0.11** | **-0.12** | **-0.12** | **-0.12** | **-0.08** | **-0.11** | **-0.10** | **-0.07** | **-0.06** | -0.04 | **-0.07** |  | **0.07** | 0.04 | **0.07** |
|  | FRA | **-0.10** | **-0.12** | **-0.11** | **-0.12** | **-0.10** | **-0.11** | **-0.12** | **-0.10** | -0.04 | -0.05 | **-0.06** | -0.03 | -0.04 |  | **0.07** | **0.09** |
|  | DEU | **-0.05** | **-0.06** | **-0.07** | **-0.06** | -0.04 | -0.02 | **-0.09** | **-0.06** | -0.04 | -0.04 | -0.04 | -0.05 | -0.04 | **-0.05** |  | 0.02 |
|  | CHE | **-0.05** | **-0.07** | **-0.07** | **-0.07** | **-0.06** | -0.04 | **-0.08** | -0.03 | **-0.06** | **-0.07** | **-0.08** | **-0.09** | **-0.08** | **-0.05** | **-0.05** |  |

^1^ Differences greater than 0.05 are reported in bold. ^2^ Population: CZE = Czech Republic, DFS = Denmark, Finland and Sweden, ESP = Spain, GBR = Great Britain, IRL = Ireland, FRA = France, DEU = Germany, CHE = Switzerland. HM = herds selected based on harmonic mean of sire’s progeny size, ALL = all data.

Table S8. Average differences in GS between each random subset of FRA-herds of RND and ALL^1^.

| Subset n. | Average GS difference |
| --- | --- |
| 1 | -0.0044 |
| *2* | *-0.0012* |
| 3 | -0.0050 |
| 4 | -0.0035 |
| 5 | -0.0020 |
| 6 | -0.0028 |
| 7 | -0.0030 |
| 8 | -0.0019 |
| 9 | -0.0023 |
| 10 | -0.0021 |
| 11 | -0.0038 |
| 12 | -0.0032 |
| 13 | -0.0018 |
| 14 | -0.0045 |
| *15* | *-0.0007* |
| 16 | -0.0027 |
| 17 | -0.0036 |
| 18 | -0.0052 |
| 19 | -0.0037 |
| *20* | *-0.0008* |

^1^ The 3 analysed subsets are reported in italic.
