# Supplementary material for: Impact of sub-setting the data of the main Limousin beef cattle population on the estimates of across-country genetic correlations
Source: Genet Sel Evol. 2020 Jun 23;52:32. doi: 10.1186/s12711-020-00551-9 (PMC7310393; doi:10.1186/s12711-020-00551-9)
Supplement: Supplementary file 2 — Additional file 2: Figure S1. Adjusted Number of Populations (AN_POP) for all common bulls in function of their year of birth and country of first registration. Figure S2. Estimated (co)variance components per population across different scenarios. Figure S3. Estimated random contemporary group and sire by herd interaction variances across different scenarios. [file 12711_2020_551_MOESM2_ESM.docx]

# Additional file 2: Figures S1-S3


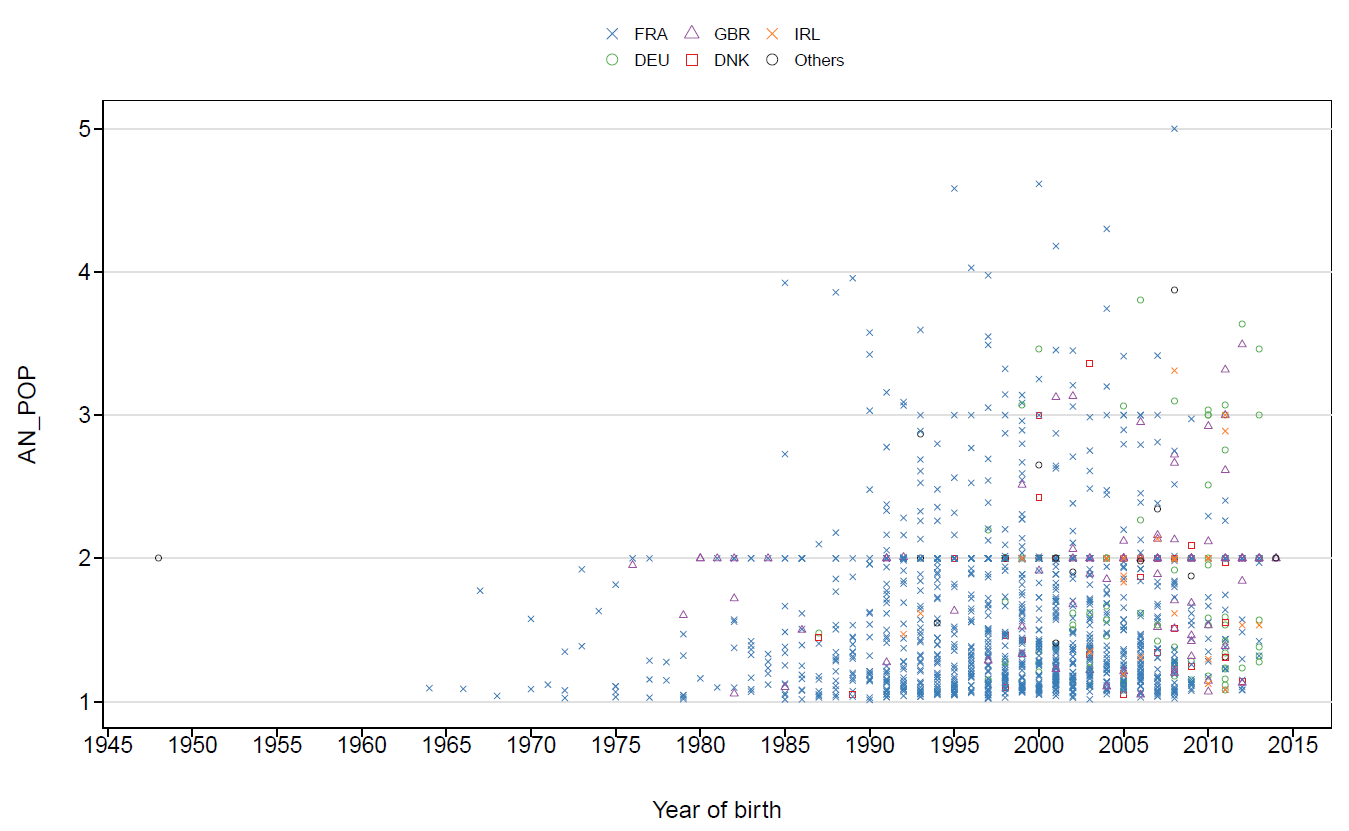


Figure S1. Adjusted Number of Populations (AN_POP) for all common bulls in function of their year of birth and country of first registration. DEU = Germany, DNK = Denmark, FRA = France, GBR = Great Britain, IRL = Ireland.


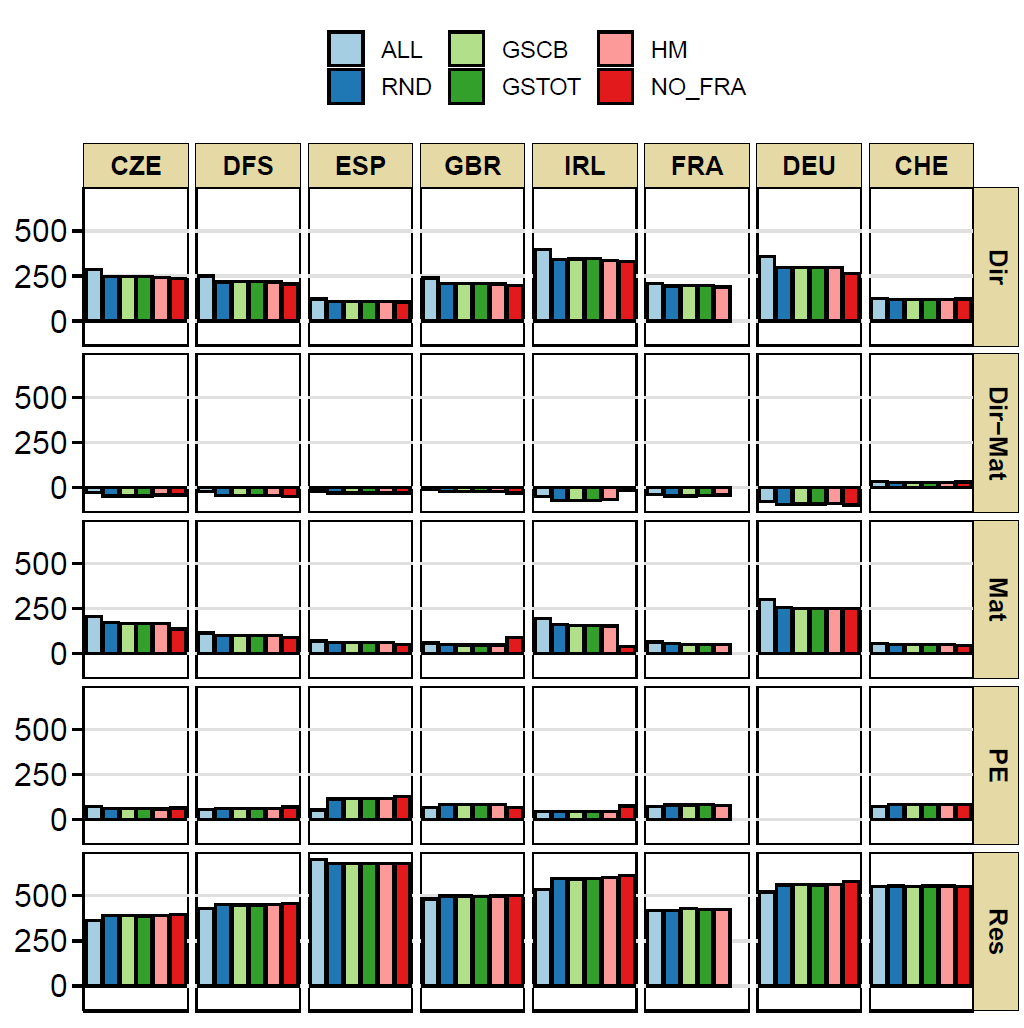


Figure S2. Estimated (co)variance components per population across different scenarios. ALL = all data, RND = herds selected randomly, GSCB = herds selected based on genetic similarity considering common bulls, GSTOT = herds selected based on genetic similarity considering common bulls and common maternal grandsires, HM = herds selected based on harmonic mean of sire’s progeny size, NO_FRA = FRA population not included in the analysis, CZE = Czech Republic, DFS = Denmark, Finland and Sweden, ESP = Spain, GBR = Great Britain, IRL = Ireland, FRA = France, DEU = Germany, CHE = Switzerland, Dir = direct genetic variance, Mat = maternal genetic variance, Dir-Mat = direct-maternal genetic covariance, PE = maternal permanent environmental variance, Res = residual variance.


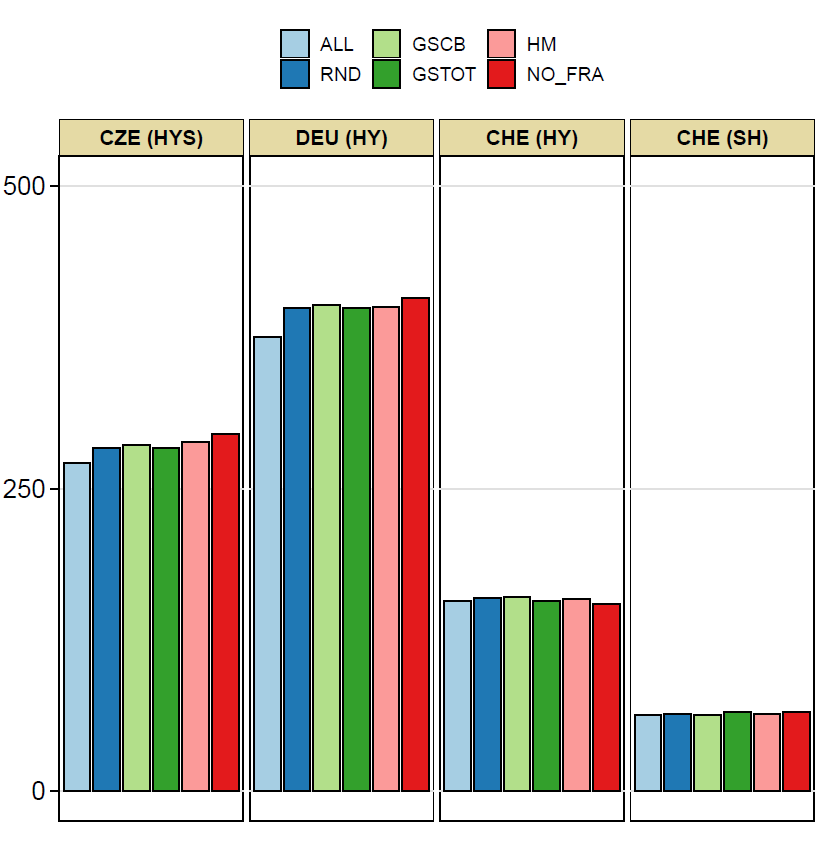


Figure S3. Estimated random contemporary group and sire by herd interaction variances across different scenarios. ALL = all data, RND = herds selected randomly, GSCB = herds selected based on genetic similarity considering common bulls, GSTOT = herds selected based on genetic similarity considering common bulls and common maternal grandsires, HM = herds selected based on harmonic mean of sire’s progeny size, NO_FRA = FRA population not included in the analysis, CHE = Switzerland, CZE = Czech Republic, DEU = Germany, HYS = Herd-Year-Season, HY = Herd-Year, SH = Sire by Herd interaction.
